# Supplementary figures and images for: A multi-pathogen behavioral exposure model for young children playing in public spaces in developing communities
Source: PLoS Negl Trop Dis. 2024 Oct 8;18(10):e0012564. doi: 10.1371/journal.pntd.0012564 (PMC11554075; doi:10.1371/journal.pntd.0012564)

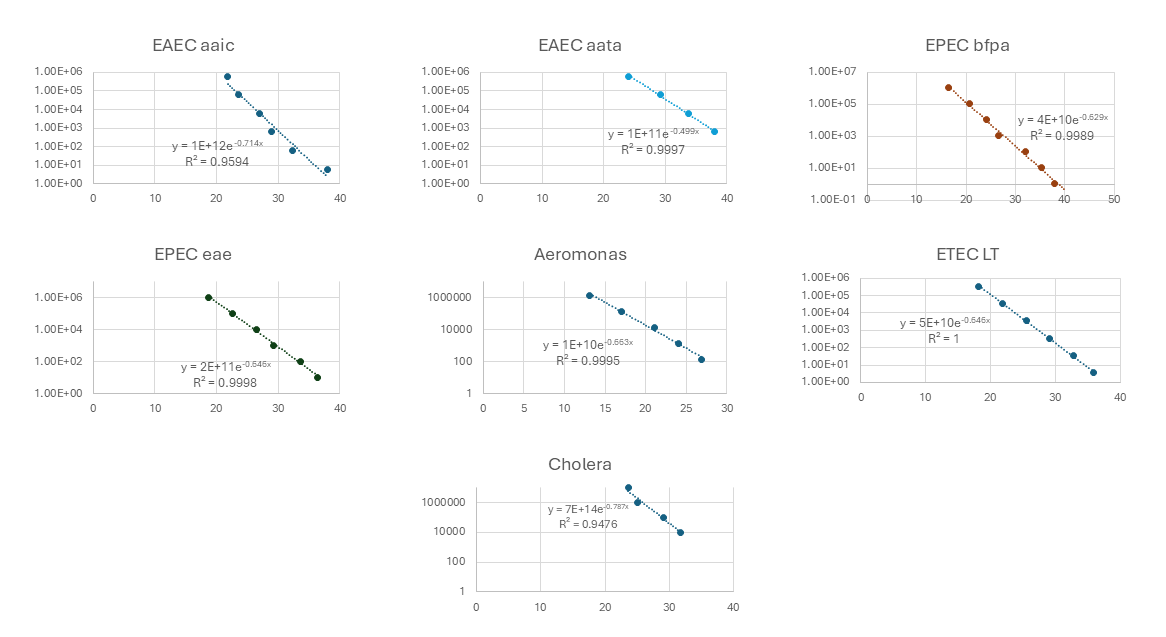

Supplement: S1 Fig — (TIF) [file pntd.0012564.s001.tif]
