# Supplementary material for: A multi-pathogen behavioral exposure model for young children playing in public spaces in developing communities
Source: PLoS Negl Trop Dis. 2024 Oct 8;18(10):e0012564. doi: 10.1371/journal.pntd.0012564 (PMC11554075; doi:10.1371/journal.pntd.0012564)
Supplement: S1 Text — (PDF) [file pntd.0012564.s003.pdf]

Statistical model code, explanation, and additional results

Preamble

Load in required packages:

```
library(magrittr)
library(bayesplot)
library(flextable)
library(corrplot)
library(truncdist)
library(rstanarm)
library(brms)
library(triangulr)
library(tidyverse)
library(ggplot2)
library(viridis)
```

Read in data:

```
soil = read_csv("https://myweb.uiowa.edu/dksewell/public/haiti-
     soil.csv")
behavior = read_csv("https://myweb.uiowa.edu/dksewell/public/haiti-
     behavioral.csv")
```

Look at summary statistics

```
soil %>%
  select(-SiteID) %>%
  pivot_longer(cols = everything(),
               names_to = "Pathogen",
               values_to = "log10conc") %>%
  group_by(Pathogen) %>%
  summarize(Detection = sum(!is.na(log10conc)),
            `Mean concentration` = mean(10^na.omit(log10conc)),
            `Median concentration` = median(10^na.omit(log10conc)),
            `Min concentration` = min(10^na.omit(log10conc)),
            `Max concentration` = max(10^na.omit(log10conc))) %>%
  flextable() %>%
  colformat_double(digits = 0)
```

Pathogen	Detection	Mean concentration	Median concentration	Min concentration	Max concentration
Aeromonas	5	5,478	130	85	19,938
Cholera	9	484,112	477,807	111,195	873,786
EAEC aaic	13	478,779	934	455	6,200,779
EAEC aata	2	206,474	206,474	119,515	293,432
EPEC bfpa	14	10,996	2,368	210	75,628
EPEC eae	38	306,567	3,928	689	6,947,445
ETEC LT	22	18,851	644	79	385,419

```
behavior %>%
  group_by(age) %>%
  summarize(`Total time observed` = sum(time_min),
            `Geophagy/hr` = sum(geophagy)/sum(time_min) * 60,
           `Mouth-to-hand/hr` = sum(mouth_to_hand)/sum(time_min)*60,
      `Mouth-to-object/hr` =sum(mouth_to_object)/sum(time_min)*60,
      `Hand-to-object/hr` = sum(hand_to_object)/sum(time_min)*60,
      `Hand-to-soil/hr` = sum(hand_to_soil)/sum(time_min)*60) %>%
  flextable() %>%
  colformat_double(digits = 1)
```

age	Total time observed	Geophagy/hr	Mouth-to-hand/hr	Mouth-to-object/hr	Hand-to-object/hr	Hand-to-soil/hr
I	356.4	0.7	17.5	4.4	20.2	10.4
T	1,069.6	0.1	11.2	2.2	19.2	9.3
C	1,859.9	0.1	8.5	1.9	26.6	12.0

Soil pathogen concentration modeling

Letting z_{ij} denote the binary indicator of the presence of pathogen j for subject i and y_{ij} its log10 concentration for i=1,…,N and j=1,…,J, we wish to model

$$\pi\left(\{z_{ij}, y_{ij}\}_{j=1}^{J}\right).$$

We fix $\pi(y_{ij}|z_{ij} = 0) = \delta_0(y_{ij})$, i.e., the log_10 concentration distribution is a point mass on 0 if not present. Zero is selected for computational convenience and is suitable since all $y_{ij}|z_{ij} = 1 > 0$ in our dataset.

We accomplish this joint modeling through telescoping out the likelihood:

$$\pi\left(\{z_{ij}, y_{ij}\}_{j=1}^{J}\right) = \pi(z_{i1})\pi(y_{i1}|z_{i1}) \prod_{j=2}^{J} \pi(z_{ij}|y_{i1}, \dots, y_{i(j-1)})\pi(y_{ij}|z_{ij}, y_{i1}, \dots, y_{i(j-1)}).$$

Note that this decomposition assumes that all the information contained in z_{ik} is also contained in y_{ik}, and so y_{ij}, $j > k$, is conditionally independent of z_{ik} given y_{ik}. We model these distributions using generalized linear models (GLMs) using $\{y_{ik}\}k < j$ as covariates for predicting both z_{ij} and y_{ij}.

Model fitting

```
# Create the "z's", i.e., the presence/absence variables, and
# set log10 conc of non-detects to zero.
soil %<>%
  select(-SiteID) %>%
  mutate_all(function(x) ifelse(is.na(x),0,x)) %>%
  bind_cols(soil %>%
              select(-SiteID) %>%
              mutate_all(function(x) ifelse(is.na(x),0, 1)) %>%
              rename_all(function(x) paste0(x,"_binary"))) %>%
  janitor::clean_names()

# Get pathogen names
path_names =
  soil %>%
  select(-contains("_binary")) %>%
  colnames()

# Create objects to store model fits
z_fits =
  y_fits =
  list()

# Look at order of prevalence
n_pos =
  soil %>%
  select(all_of(path_names)) %>%
  as.matrix() %>%
  apply(2,function(x) sum(x > 0))
```

EAEC aata

EAEC aata only had 2 positive detects, not enough to statistically identify associations with any other pathogens.

```r
z_fits$eaec_aata =
  stan_glm(eaec_aata_binary ~ 1,
           family = binomial(),
           data = soil,
           iter = 10000,
           seed = 2024)

summary(z_fits$eaec_aata)
```

```
Model Info:
 function:     stan_glm
 family:       binomial [logit]
 formula:      eaec_aata_binary ~ 1
 algorithm:    sampling
 sample:       20000 (posterior sample size)
 priors:       see help('prior_summary')
 observations: 79
 predictors:   1

Estimates:
              mean   sd   10%   50%   90%
(Intercept) -3.6    0.6 -4.4  -3.5  -2.8

Fit Diagnostics:
           mean   sd   10%   50%   90%
mean_PPD 0.0    0.0  0.0   0.0   0.1

The mean_ppd is the sample average posterior predictive
distribution of the outcome variable (for details see
help('summary.stanreg')).

MCMC diagnostics
              mcse Rhat n_eff
(Intercept)   0.0  1.0  6006
mean_PPD      0.0  1.0  9916
log-posterior 0.0  1.0  5438

For each parameter, mcse is Monte Carlo standard error, n_eff is
a crude measure of effective sample size, and Rhat is the
potential scale reduction factor on split chains (at convergence
Rhat=1).
```

```r
mcmc_trace(z_fits$eaec_aata)
```

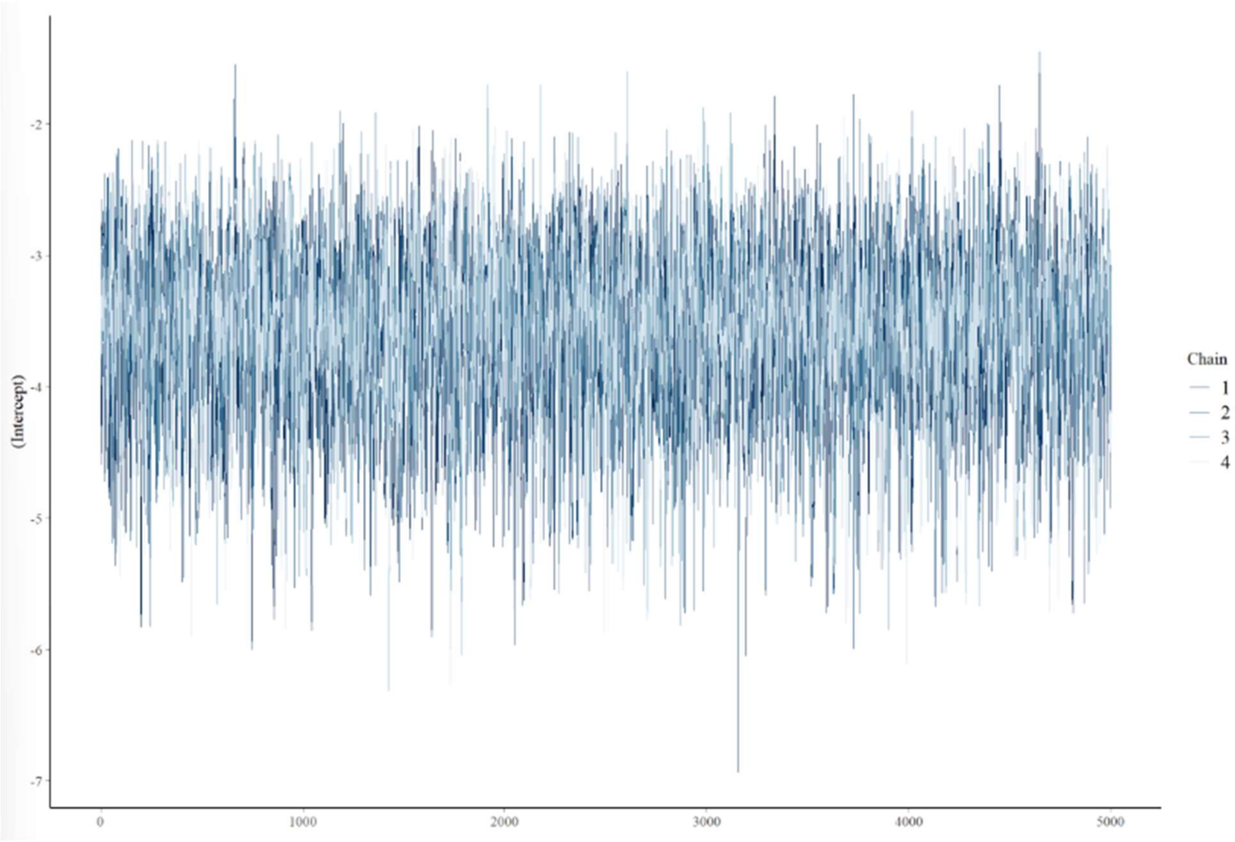


```
y_fits$eaec_aata =
  stan_glm(eaec_aata ~ 1,
           family = gaussian(),
           data =
             soil %>%
             filter(eaec_aata_binary == 1),
           iter = 10000,
           seed = 2024)

summary(y_fits$eaec_aata)
```

```
Model Info:
 function:     stan_glm
 family:       gaussian [identity]
 formula:      eaec_aata ~ 1
 algorithm:    sampling
 sample:       20000 (posterior sample size)
 priors:       see help('prior_summary')
 observations: 2
 predictors:   1

Estimates:
              mean   sd   10%   50%   90%
```

```
(Intercept) 5.3    0.2  5.0   5.3   5.5
sigma       0.3    0.2  0.2   0.3   0.6

Fit Diagnostics:
           mean   sd   10%   50%   90%
mean_PPD 5.3    0.4  4.9   5.3   5.7

The mean_ppd is the sample average posterior predictive
distribution of the outcome variable (for details see
help('summary.stanreg')).

MCMC diagnostics
              mcse Rhat n_eff
(Intercept)   0.0  1.0  5237
sigma         0.0  1.0  5816
mean_PPD      0.0  1.0  9832
log-posterior 0.0  1.0  4387

For each parameter, mcse is Monte Carlo standard error, n_eff is
a crude measure of effective sample size, and Rhat is the
potential scale reduction factor on split chains (at convergence
Rhat=1).
```

```
mcmc_trace(y_fits$eaec_aata)
```

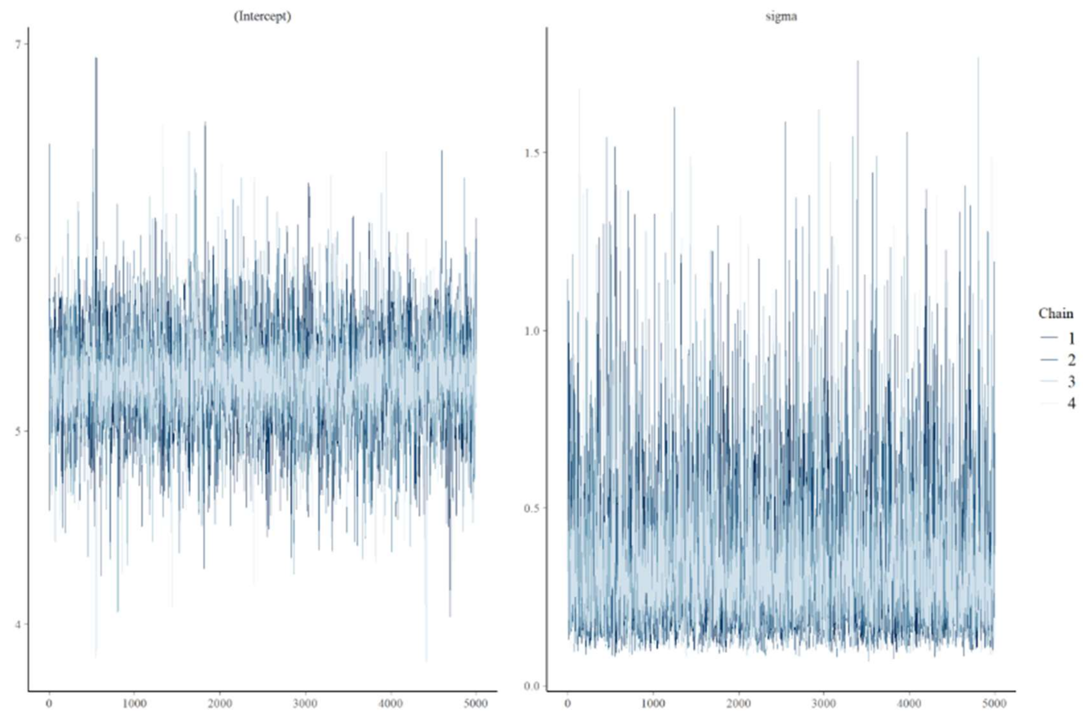

Aeromonas

```
z_fits$aeromonas =
  stan_glm(aeromonas_binary ~ 1,
           family = binomial(),
           data = soil,
           iter = 10000,
           seed = 2024)

summary(z_fits$aeromonas)
```

```
Model Info:
 function:     stan_glm
 family:       binomial [logit]
 formula:      aeromonas_binary ~ 1
 algorithm:    sampling
 sample:       20000 (posterior sample size)
 priors:       see help('prior_summary')
 observations: 79
 predictors:   1

Estimates:
              mean   sd   10%   50%   90%
(Intercept) -2.7    0.4 -3.3  -2.7  -2.1

Fit Diagnostics:
           mean   sd   10%   50%   90%
mean_PPD 0.1    0.0  0.0   0.1   0.1

The mean_ppd is the sample average posterior predictive
distribution of the outcome variable (for details see
help('summary.stanreg')).

MCMC diagnostics
              mcse Rhat n_eff
(Intercept)   0.0  1.0  5303
mean_PPD      0.0  1.0  9169
log-posterior 0.0  1.0  5069

For each parameter, mcse is Monte Carlo standard error, n_eff is
a crude measure of effective sample size, and Rhat is the
potential scale reduction factor on split chains (at convergence
Rhat=1).
```

```
mcmc_trace(z_fits$aeromonas)
```

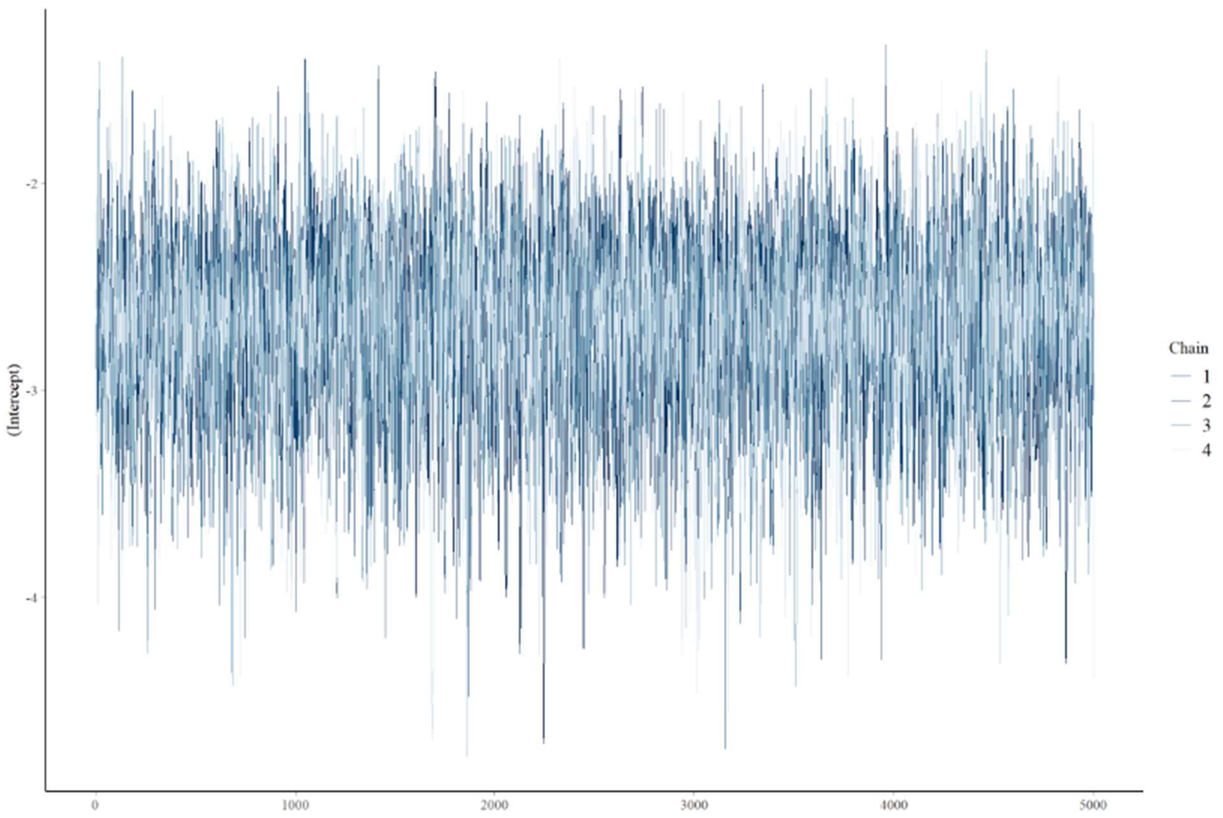


```
y_fits$aeromonas =
  stan_glm(aeromonas ~ 1,
           family = gaussian(),
           data =
             soil %>%
             filter(aeromonas_binary == 1),
           iter = 10000,
           seed = 2024)

summary(y_fits$aeromonas)
```

```
Model Info:
 function:     stan_glm
 family:       gaussian [identity]
 formula:      aeromonas ~ 1
 algorithm:    sampling
 sample:       20000 (posterior sample size)
 priors:       see help('prior_summary')
 observations: 5
 predictors:   1

Estimates:
              mean   sd   10%   50%   90%
(Intercept) 2.8    0.6  2.1   2.8   3.6
```

```
sigma       1.3    0.5  0.8   1.2   1.9

Fit Diagnostics:
           mean   sd   10%   50%   90%
mean_PPD 2.8    0.9  1.8   2.8   3.9

The mean_ppd is the sample average posterior predictive
distribution of the outcome variable (for details see
help('summary.stanreg')).

MCMC diagnostics
              mcse Rhat n_eff
(Intercept)   0.0  1.0   7232
sigma         0.0  1.0   6639
mean_PPD      0.0  1.0  11282
log-posterior 0.0  1.0   5094

For each parameter, mcse is Monte Carlo standard error, n_eff is
a crude measure of effective sample size, and Rhat is the
potential scale reduction factor on split chains (at convergence
Rhat=1).
```

```r
mcmc_trace(y_fits$aeromonas)
```

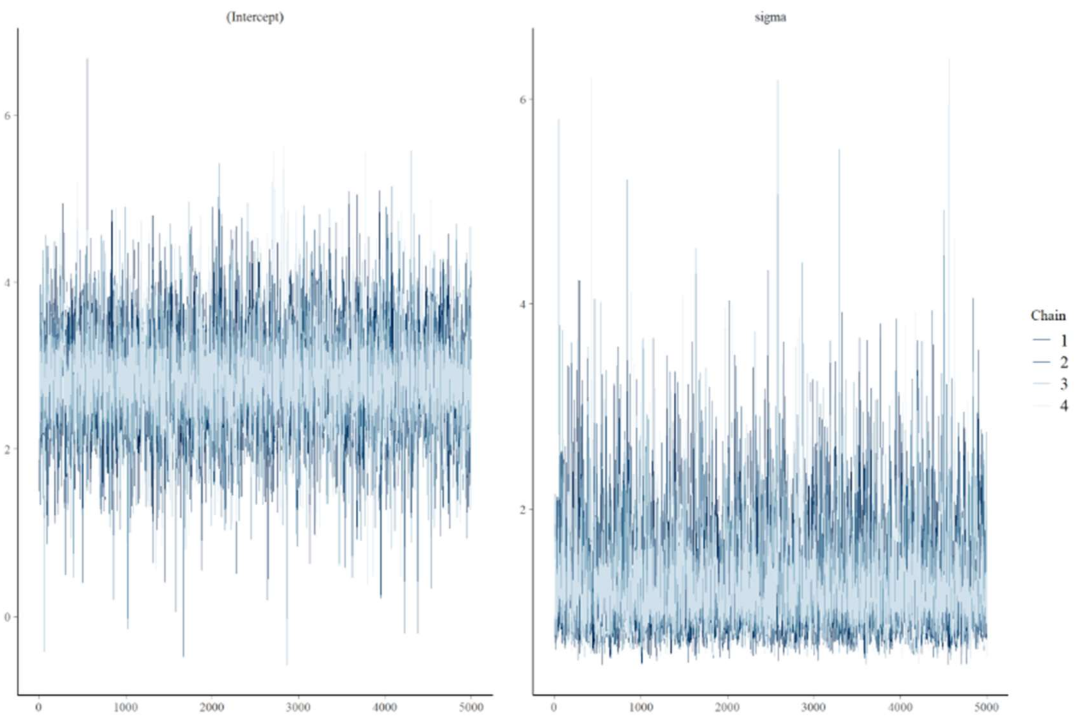

Cholera

```r
z_fits$cholera =
  stan_glm(cholera_binary ~ aeromonas,
           family = binomial(),
           data = soil,
           iter = 10000,
           seed = 2024)

summary(z_fits$cholera)
```

```
Model Info:
 function:     stan_glm
 family:       binomial [logit]
 formula:      cholera_binary ~ aeromonas
 algorithm:    sampling
 sample:       20000 (posterior sample size)
 priors:       see help('prior_summary')
 observations: 79
 predictors:   2

Estimates:
              mean   sd   10%   50%   90%
(Intercept) -2.3    0.4 -2.9  -2.3  -1.8
aeromonas    0.8    0.4  0.4   0.8   1.3

Fit Diagnostics:
           mean   sd   10%   50%   90%
mean_PPD 0.1    0.0  0.1   0.1   0.2

The mean_ppd is the sample average posterior predictive
distribution of the outcome variable (for details see
help('summary.stanreg')).

MCMC diagnostics
              mcse Rhat n_eff
(Intercept)   0.0  1.0  11516
aeromonas     0.0  1.0  10880
mean_PPD      0.0  1.0  15621
log-posterior 0.0  1.0   7219

For each parameter, mcse is Monte Carlo standard error, n_eff is
a crude measure of effective sample size, and Rhat is the
potential scale reduction factor on split chains (at convergence
Rhat=1).
```

```r
mcmc_trace(z_fits$cholera)
```

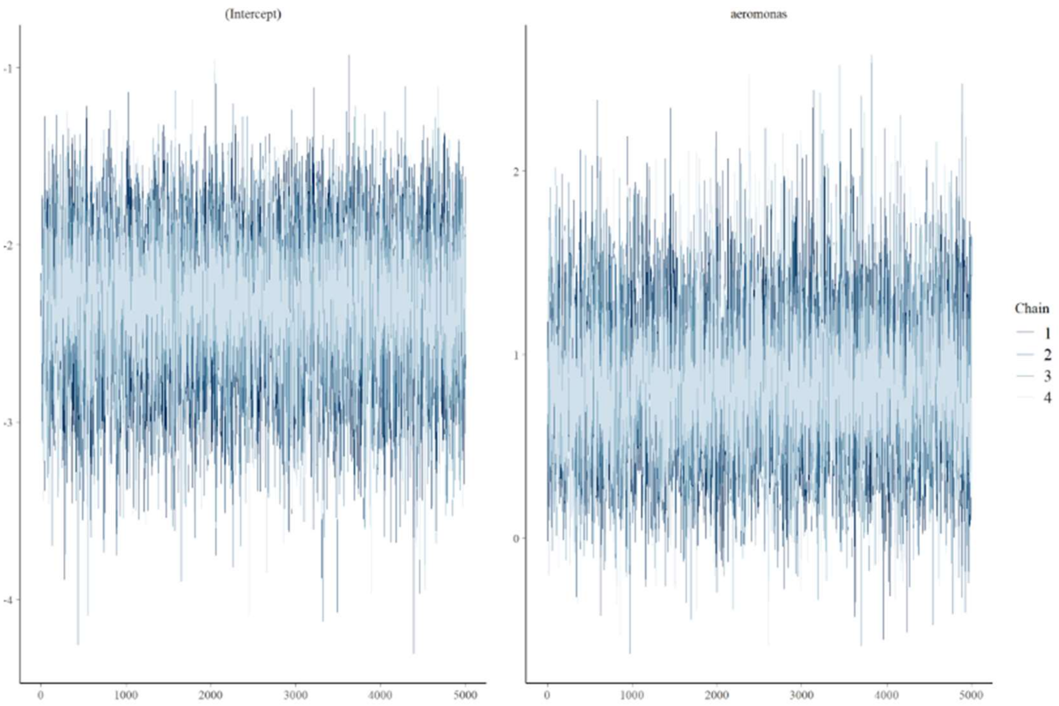


```r
y_fits$cholera =
  stan_glm(cholera ~ aeromonas,
           family = gaussian(),
           data =
             soil %>%
             filter(cholera_binary == 1),
           iter = 10000,
           seed = 2024)

summary(y_fits$cholera)
```

```
Model Info:
 function:     stan_glm
 family:       gaussian [identity]
 formula:      cholera ~ aeromonas
 algorithm:    sampling
 sample:       20000 (posterior sample size)
 priors:       see help('prior_summary')
 observations: 9
 predictors:   2

Estimates:
              mean   sd   10%   50%   90%
(Intercept)  5.5    0.2  5.3   5.5   5.7
```

```
aeromonas     0.0    0.1 -0.1   0.0   0.2
sigma         0.4    0.1  0.3   0.4   0.6

Fit Diagnostics:
           mean   sd   10%   50%   90%
mean_PPD 5.6    0.2  5.3   5.6   5.8

The mean_ppd is the sample average posterior predictive
distribution of the outcome variable (for details see
help('summary.stanreg')).

MCMC diagnostics
              mcse Rhat n_eff
(Intercept)   0.0  1.0  12120
aeromonas     0.0  1.0  13085
sigma         0.0  1.0  10149
mean_PPD      0.0  1.0  14891
log-posterior 0.0  1.0   6520

For each parameter, mcse is Monte Carlo standard error, n_eff is
a crude measure of effective sample size, and Rhat is the
potential scale reduction factor on split chains (at convergence
Rhat=1).
```

```
mcmc_trace(y_fits$cholera)
```

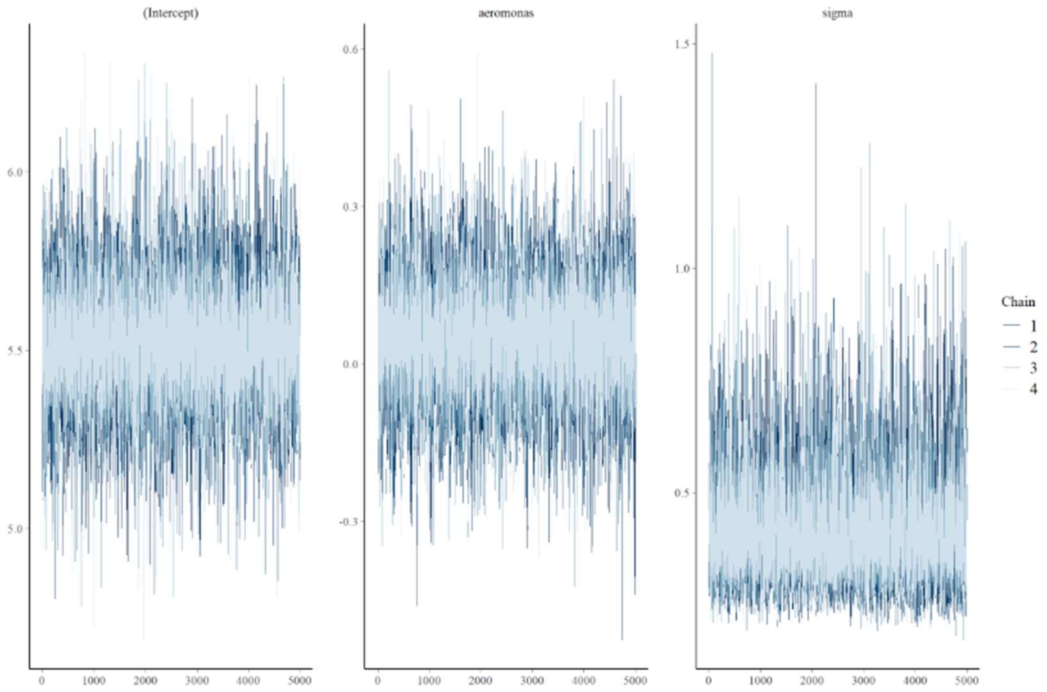

EAEC aaic

```
z_fits$eaec_aaic =
  stan_glm(eaec_aaic_binary ~ aeromonas + cholera,
           family = binomial(),
           data = soil,
           iter = 10000,
           seed = 2024)

summary(z_fits$eaec_aaic)
```

```
Model Info:
 function:     stan_glm
 family:       binomial [logit]
 formula:      eaec_aaic_binary ~ aeromonas + cholera
 algorithm:    sampling
 sample:       20000 (posterior sample size)
 priors:       see help('prior_summary')
 observations: 79
 predictors:   3

Estimates:
              mean   sd   10%   50%   90%
(Intercept) -1.8    0.3 -2.2  -1.8  -1.4
aeromonas   -0.5    0.6 -1.3  -0.4   0.2
cholera      0.2    0.2  0.0   0.2   0.4

Fit Diagnostics:
           mean   sd   10%   50%   90%
mean_PPD 0.2    0.1  0.1   0.2   0.2

The mean_ppd is the sample average posterior predictive
distribution of the outcome variable (for details see
help('summary.stanreg')).

MCMC diagnostics
              mcse Rhat n_eff
(Intercept)   0.0  1.0  15782
aeromonas     0.0  1.0  10597
cholera       0.0  1.0  14452
mean_PPD      0.0  1.0  18823
log-posterior 0.0  1.0   6963

For each parameter, mcse is Monte Carlo standard error, n_eff is
a crude measure of effective sample size, and Rhat is the
```

potential scale reduction factor on split chains (at convergence Rhat=1).

```
mcmc_trace(y_fits$cholera)
```

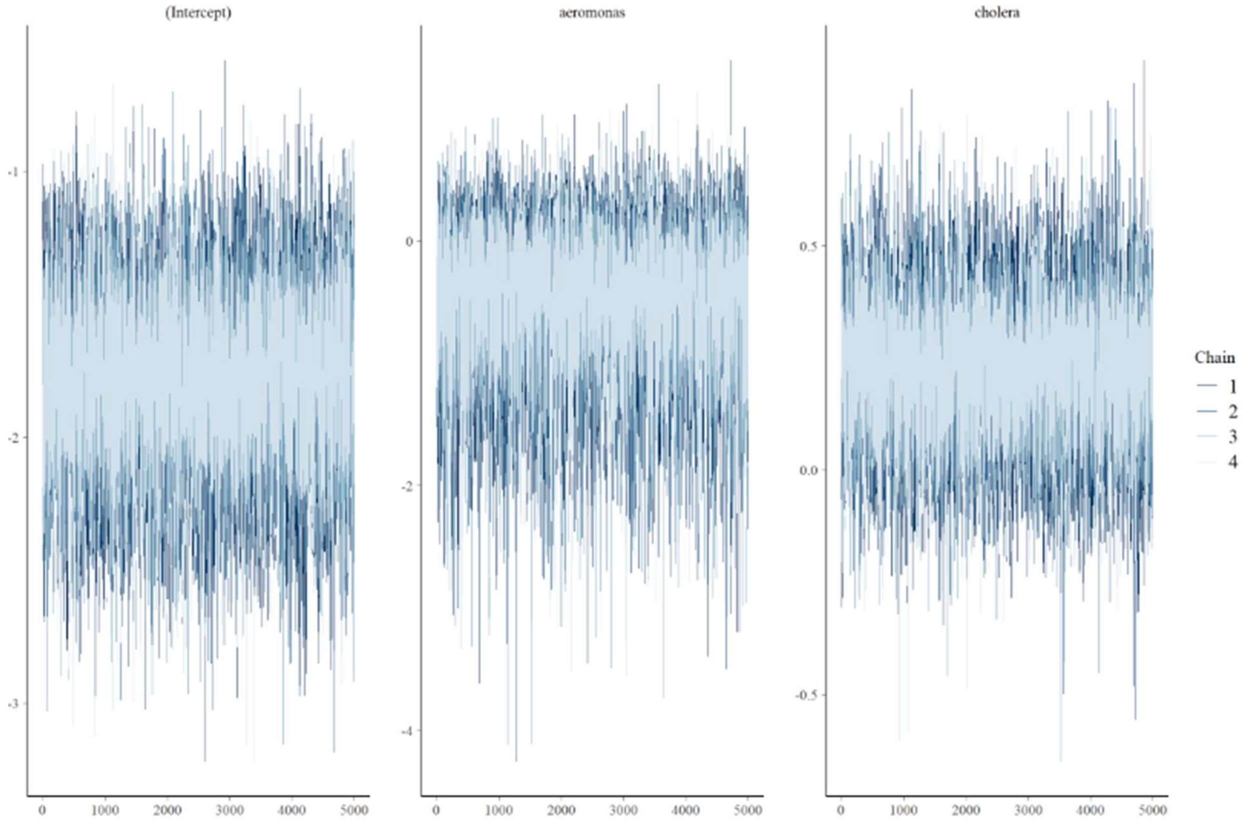


```
y_fits$eaec_aaic =
  stan_glm(eaec_aaic ~ aeromonas + cholera,
           family = gaussian(),
           data =
             soil %>%
             filter(eaec_aaic_binary == 1),
           iter = 10000,
           seed = 2024)

summary(y_fits$eaec_aaic)
```

```
Model Info:
 function:     stan_glm
 family:       gaussian [identity]
 formula:      eaec_aaic ~ aeromonas + cholera
 algorithm:    sampling
 sample:       20000 (posterior sample size)
 priors:       see help('prior_summary')
```

```
 observations: 13
 predictors:   3

Estimates:
              mean   sd   10%   50%   90%
(Intercept)  3.1    0.1  2.9   3.1   3.3  
aeromonas    1.7    0.3  1.4   1.7   2.1  
cholera      0.0    0.1 -0.1   0.0   0.1  
sigma        0.4    0.1  0.3   0.4   0.6  

Fit Diagnostics:
           mean   sd   10%   50%   90%
mean_PPD 3.4    0.2  3.2   3.4   3.6  

The mean_ppd is the sample average posterior predictive
distribution of the outcome variable (for details see
help('summary.stanreg')).

MCMC diagnostics
              mcse Rhat n_eff
(Intercept)   0.0  1.0  16470
aeromonas     0.0  1.0  11946
cholera       0.0  1.0  11931
sigma         0.0  1.0   9782
mean_PPD      0.0  1.0  17551
log-posterior 0.0  1.0   6087

For each parameter, mcse is Monte Carlo standard error, n_eff is
a crude measure of effective sample size, and Rhat is the
potential scale reduction factor on split chains (at convergence
Rhat=1).
```

```r
mcmc_trace(y_fits$eaec_aaic)
```

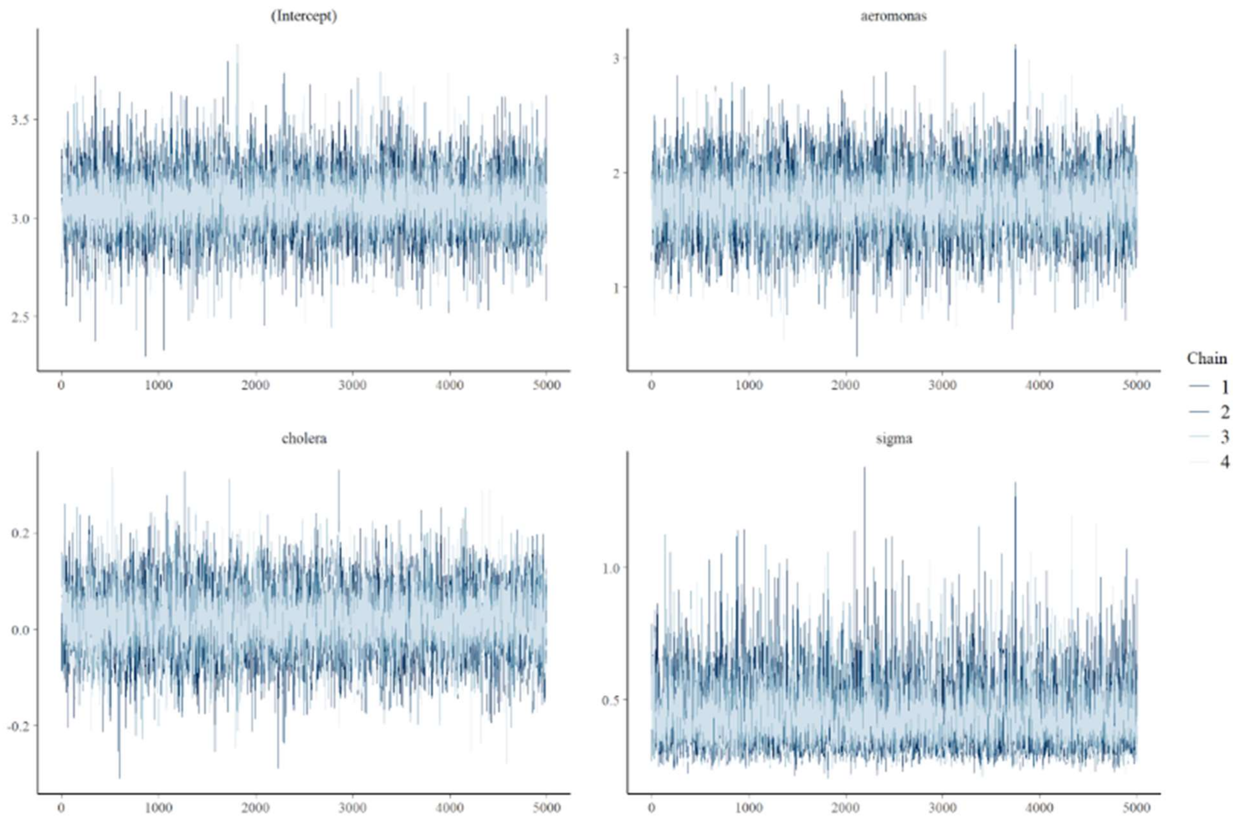

<u>EPEC bfpa</u>

```
z_fits$epec_bfpa =
  stan_glm(epec_bfpa_binary ~ aeromonas + cholera + eaec_aaic,
           family = binomial(),
           data = soil,
           iter = 10000,
           seed = 2024)

summary(z_fits$epec_bfpa)
```

```
Model Info:
 function:     stan_glm
 family:       binomial [logit]
 formula:      epec_bfpa_binary ~ aeromonas + cholera +
eaec_aaic
 algorithm:    sampling
 sample:       20000 (posterior sample size)
 priors:       see help('prior_summary')
 observations: 79
 predictors:   4

Estimates:
```

```
              mean   sd   10%   50%   90%
(Intercept) -2.3    0.4 -2.9  -2.3  -1.8
aeromonas   -0.8    0.9 -2.0  -0.6   0.3
cholera     -0.2    0.3 -0.5  -0.2   0.1
eaec_aaic    1.0    0.2  0.7   1.0   1.3

Fit Diagnostics:
           mean   sd   10%   50%   90%
mean_PPD 0.2    0.1  0.1   0.2   0.3

The mean_ppd is the sample average posterior predictive
distribution of the outcome variable (for details see
help('summary.stanreg')).

MCMC diagnostics
              mcse Rhat n_eff
(Intercept)   0.0  1.0  12700
aeromonas     0.0  1.0   9705
cholera       0.0  1.0  11473
eaec_aaic     0.0  1.0  10589
mean_PPD      0.0  1.0  19332
log-posterior 0.0  1.0   7017

For each parameter, mcse is Monte Carlo standard error, n_eff is
a crude measure of effective sample size, and Rhat is the
potential scale reduction factor on split chains (at convergence
Rhat=1).
```

```
mcmc_trace(z_fits$epec_bfpa)
```

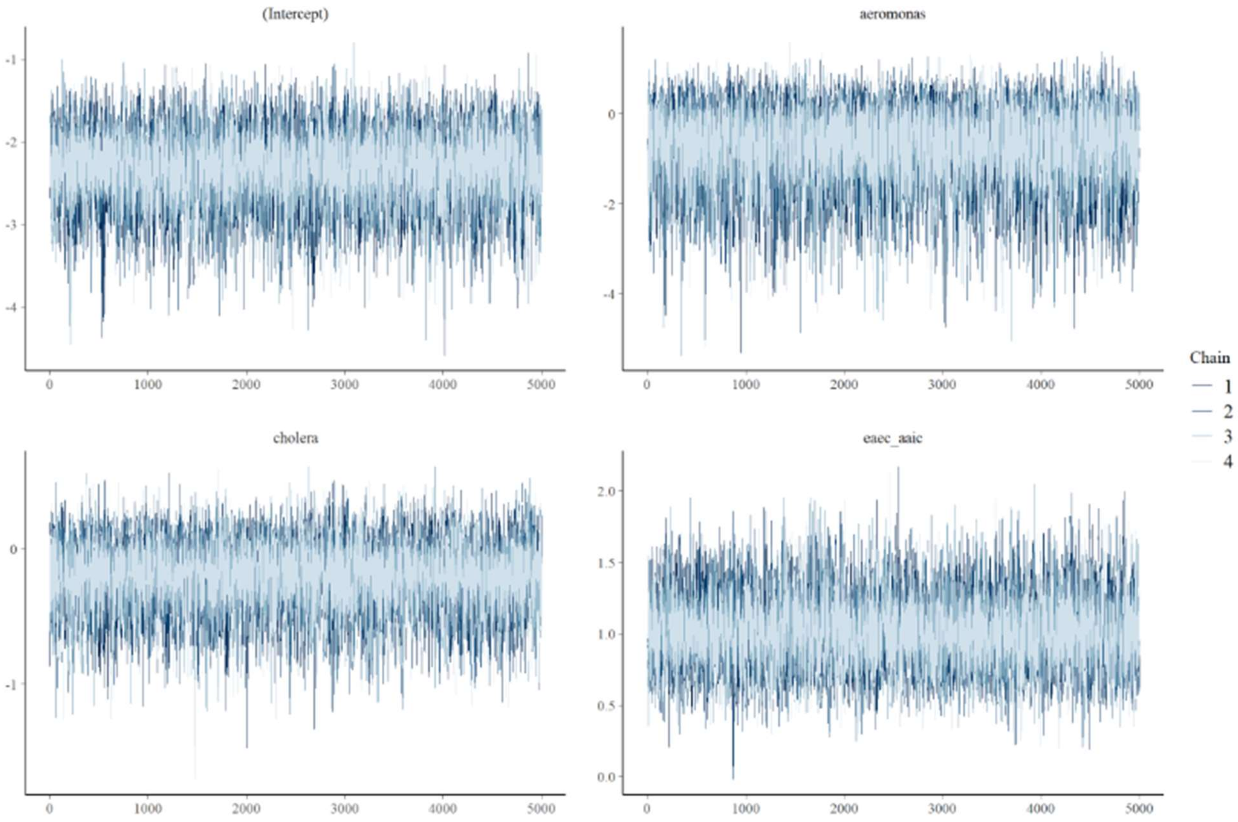


```r
y_fits$epec_bfpa =
  stan_glm(epec_bfpa ~ aeromonas + cholera + eaec_aaic,
           family = gaussian(),
           data =
             soil %>%
             filter(epec_bfpa_binary == 1),
           iter = 10000,
           seed = 2024)

summary(y_fits$epec_bfpa)
```

```
Model Info:
 function:     stan_glm
 family:       gaussian [identity]
 formula:      epec_bfpa ~ aeromonas + cholera + eaec_aaic
 algorithm:    sampling
 sample:       20000 (posterior sample size)
 priors:       see help('prior_summary')
 observations: 14
 predictors:   4

Estimates:
              mean   sd   10%   50%   90%
(Intercept)  2.9    0.3  2.6   2.9   3.3
```

```
aeromonas     0.8     0.5  0.1   0.8   1.5
cholera      -0.2     0.2 -0.4  -0.2   0.0
eaec_aaic     0.2     0.1  0.0   0.2   0.4
sigma         0.7     0.2  0.5   0.7   0.9

Fit Diagnostics:
           mean   sd   10%   50%   90%
mean_PPD 3.3    0.3  3.0   3.3   3.7

The mean_ppd is the sample average posterior predictive
distribution of the outcome variable (for details see
help('summary.stanreg')).

MCMC diagnostics
              mcse Rhat n_eff
(Intercept)   0.0  1.0  15608
aeromonas     0.0  1.0  13547
cholera       0.0  1.0  13215
eaec_aaic     0.0  1.0  12866
sigma         0.0  1.0   9698
mean_PPD      0.0  1.0  17347
log-posterior 0.0  1.0   5626

For each parameter, mcse is Monte Carlo standard error, n_eff is
a crude measure of effective sample size, and Rhat is the
potential scale reduction factor on split chains (at convergence
Rhat=1).
```

```r
mcmc_trace(y_fits$epec_bfpa)
```

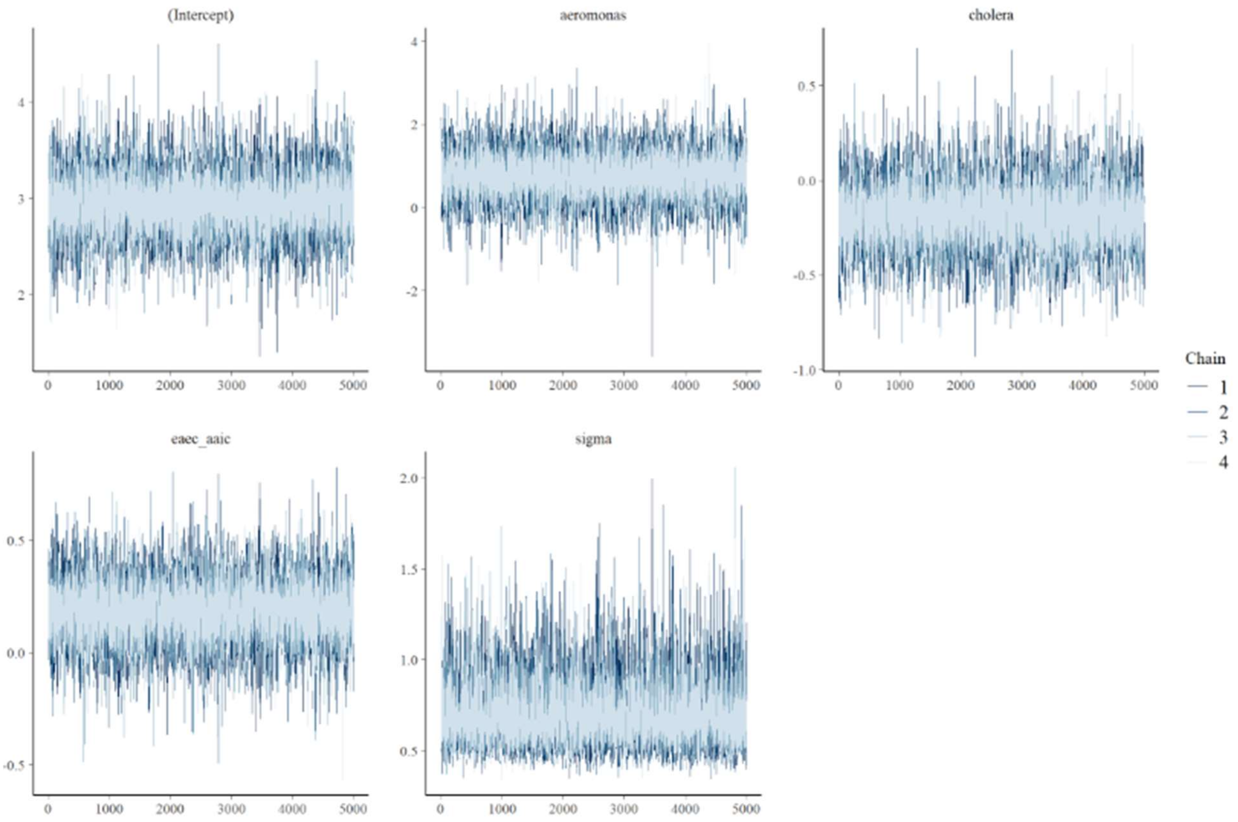

ETEC LT

```
z_fits$etec_lt =
  stan_glm(etec_lt_binary ~ aeromonas + cholera + eaec_aaic +
                epec_bfpa,
           family = binomial(),
           data = soil,
           iter = 10000,
           seed = 2024)

summary(z_fits$etec_lt)
```

```
Model Info:
 function:     stan_glm
 family:       binomial [logit]
 formula:      etec_lt_binary ~ aeromonas + cholera + eaec_aaic
+ epec_bfpa
 algorithm:    sampling
 sample:       20000 (posterior sample size)
 priors:       see help('prior_summary')
 observations: 79
 predictors:   5
```

```
Estimates:
              mean   sd   10%   50%   90%
(Intercept) -1.2    0.3 -1.6  -1.2  -0.8
aeromonas    0.2    0.4 -0.3   0.2   0.6
cholera      0.2    0.2  0.0   0.2   0.4
eaec_aaic   -0.5    0.3 -0.9  -0.5  -0.1
epec_bfpa    0.5    0.3  0.2   0.5   0.8

Fit Diagnostics:
           mean   sd   10%   50%   90%
mean_PPD 0.3    0.1  0.2   0.3   0.4

The mean_ppd is the sample average posterior predictive
distribution of the outcome variable (for details see
help('summary.stanreg')).

MCMC diagnostics
              mcse Rhat n_eff
(Intercept)   0.0  1.0  17486
aeromonas     0.0  1.0  15152
cholera       0.0  1.0  15344
eaec_aaic     0.0  1.0  11247
epec_bfpa     0.0  1.0  11941
mean_PPD      0.0  1.0  18282
log-posterior 0.0  1.0   8321

For each parameter, mcse is Monte Carlo standard error, n_eff is
a crude measure of effective sample size, and Rhat is the
potential scale reduction factor on split chains (at convergence
Rhat=1).
```

```
mcmc_trace(z_fits$etec_lt)
```

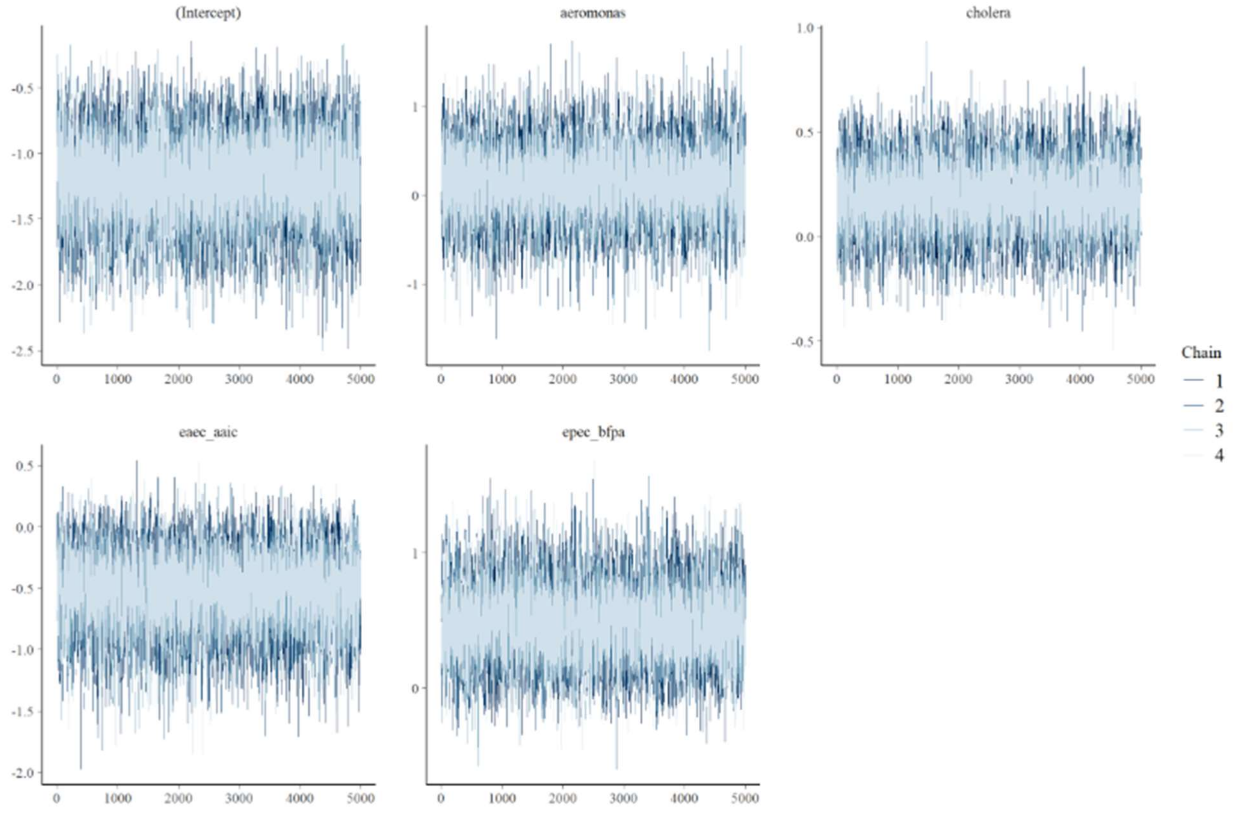


```
y_fits$etec_lt =
  stan_glm(etec_lt ~ aeromonas + cholera + eaec_aaic + epec_bfpa,
           family = gaussian(),
           data =
             soil %>%
             filter(etec_lt_binary == 1),
           iter = 10000,
           seed = 2024)

summary(y_fits$etec_lt)
```

```
Model Info:
 function:     stan_glm
 family:       gaussian [identity]
 formula:      etec_lt ~ aeromonas + cholera + eaec_aaic +
epec_bfpa
 algorithm:    sampling
 sample:       20000 (posterior sample size)
 priors:       see help('prior_summary')
 observations: 22
 predictors:   5

Estimates:
              mean   sd   10%   50%   90%
```

```
(Intercept)  2.8    0.2  2.5   2.8   3.1
aeromonas    0.2    0.2 -0.1   0.2   0.4
cholera      0.0    0.1 -0.1   0.0   0.1
eaec_aaic    0.0    0.2 -0.2   0.0   0.2
epec_bfpa    0.2    0.1  0.0   0.2   0.4
sigma        0.8    0.1  0.6   0.8   1.0

Fit Diagnostics:
           mean   sd   10%   50%   90%
mean_PPD 3.0    0.2  2.7   3.0   3.3

The mean_ppd is the sample average posterior predictive
distribution of the outcome variable (for details see
help('summary.stanreg')).

MCMC diagnostics
              mcse Rhat n_eff
(Intercept)   0.0  1.0  20560
aeromonas     0.0  1.0  21620
cholera       0.0  1.0  18880
eaec_aaic     0.0  1.0  17555
epec_bfpa     0.0  1.0  17485
sigma         0.0  1.0  15093
mean_PPD      0.0  1.0  20887
log-posterior 0.0  1.0   7426

For each parameter, mcse is Monte Carlo standard error, n_eff is
a crude measure of effective sample size, and Rhat is the
potential scale reduction factor on split chains (at convergence
Rhat=1).
```

```r
mcmc_trace(y_fits$etec_lt)
```

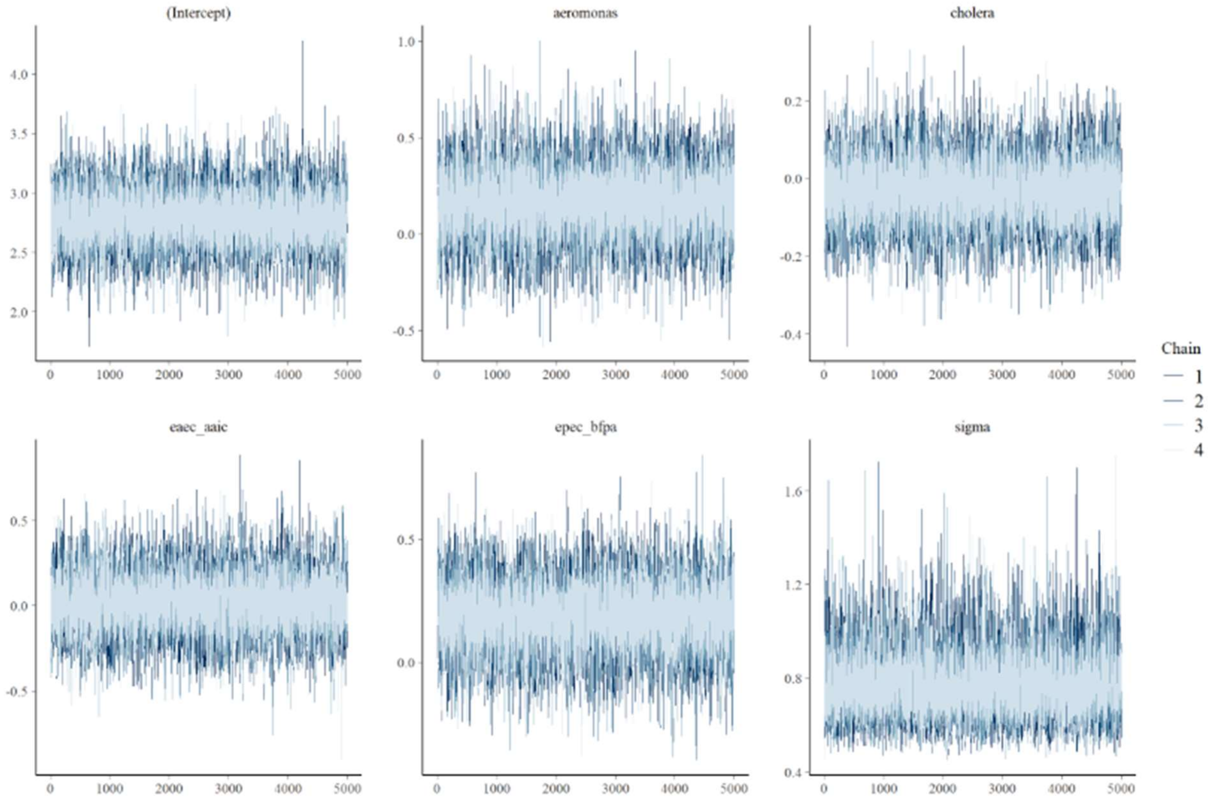

<u>EPEC eae</u>

```
z_fits$epec_eae =
  stan_glm(epec_eae_binary ~ aeromonas + cholera + eaec_aaic +
                  epec_bfpa + etec_lt,
           family = binomial(),
           data = soil,
           iter = 10000,
           seed = 2024)

summary(z_fits$epec_eae)
```

```
Model Info:
 function:     stan_glm
 family:       binomial [logit]
 formula:      epec_eae_binary ~ aeromonas + cholera + eaec_aaic
+ epec_bfpa +
       etec_lt
 algorithm:    sampling
 sample:       20000 (posterior sample size)
 priors:       see help('prior_summary')
 observations: 79
 predictors:   6
```

```
Estimates:
              mean   sd   10%   50%   90%
(Intercept) -0.7    0.3 -1.1  -0.7  -0.3
aeromonas   -0.1    0.4 -0.7  -0.1   0.4
cholera      0.3    0.2  0.1   0.3   0.6
eaec_aaic    0.1    0.3 -0.3   0.1   0.5
epec_bfpa    1.2    0.5  0.7   1.2   1.8
etec_lt      0.0    0.2 -0.3   0.0   0.2

Fit Diagnostics:
           mean   sd   10%   50%   90%
mean_PPD 0.5    0.1  0.4   0.5   0.6

The mean_ppd is the sample average posterior predictive
distribution of the outcome variable (for details see
help('summary.stanreg')).

MCMC diagnostics
              mcse Rhat n_eff
(Intercept)   0.0  1.0  30714
aeromonas     0.0  1.0  15601
cholera       0.0  1.0  15342
eaec_aaic     0.0  1.0  17128
epec_bfpa     0.0  1.0  10870
etec_lt       0.0  1.0  18482
mean_PPD      0.0  1.0  23957
log-posterior 0.0  1.0   8172

For each parameter, mcse is Monte Carlo standard error, n_eff is
a crude measure of effective sample size, and Rhat is the
potential scale reduction factor on split chains (at convergence
Rhat=1).
```

```
mcmc_trace(z_fits$epec_eae)
```

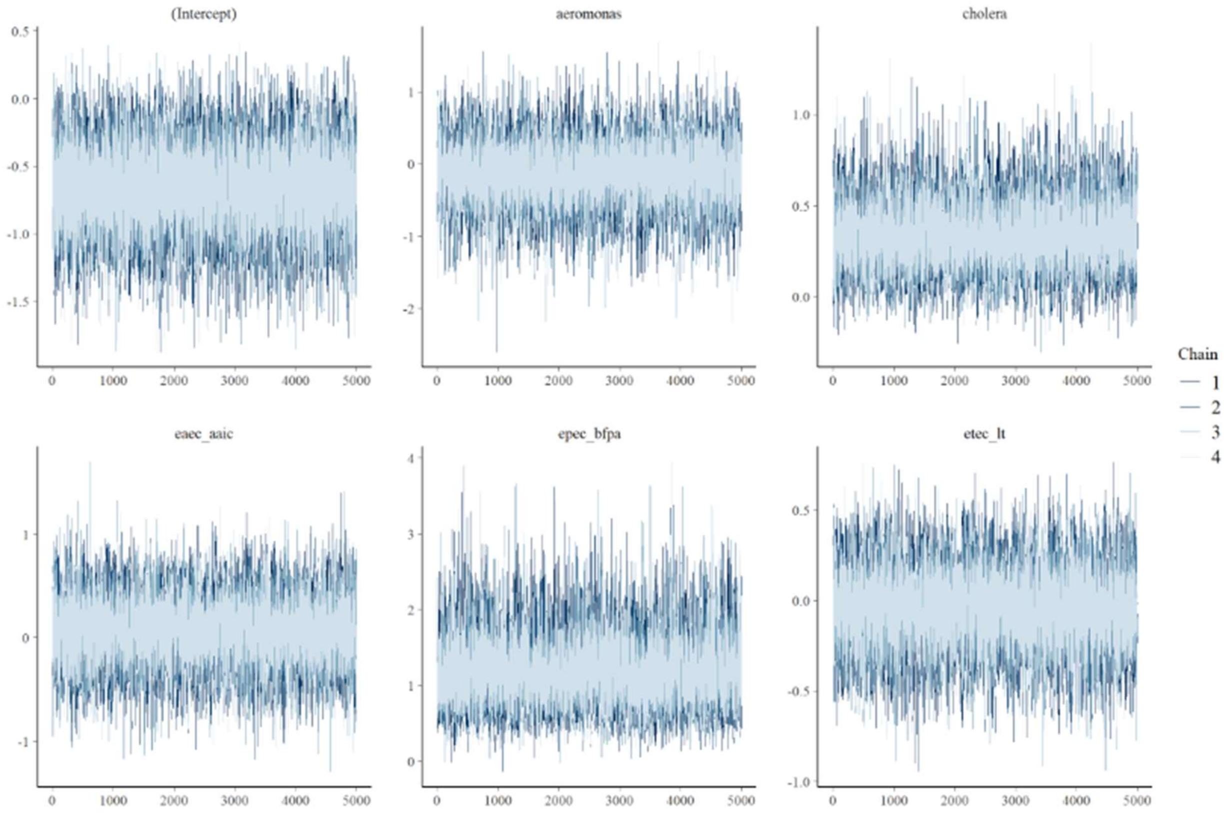


```r
y_fits$epec_eae =
  stan_glm(epec_eae ~ aeromonas + cholera + eaec_aaic + epec_bfpa +
                etec_lt,
           family = gaussian(),
           data =
             soil %>%
             filter(epec_eae_binary == 1),
           iter = 10000,
           seed = 2024)

summary(y_fits$epec_eae)
```

```
Model Info:
 function:     stan_glm
 family:       gaussian [identity]
 formula:      epec_eae ~ aeromonas + cholera + eaec_aaic +
epec_bfpa + etec_lt
 algorithm:    sampling
 sample:       20000 (posterior sample size)
 priors:       see help('prior_summary')
 observations: 38
 predictors:   6
```

```
Estimates:
              mean   sd   10%   50%   90%
(Intercept)  3.7    0.2  3.5   3.7   3.9 
aeromonas    0.1    0.2 -0.2   0.1   0.3 
cholera      0.0    0.1 -0.1   0.0   0.1 
eaec_aaic    0.1    0.1 -0.1   0.1   0.2 
epec_bfpa    0.4    0.1  0.3   0.4   0.5 
etec_lt     -0.2    0.1 -0.3  -0.2  -0.1 
sigma        0.8    0.1  0.6   0.8   0.9 

Fit Diagnostics:
           mean   sd   10%   50%   90%
mean_PPD 4.0    0.2  3.8   4.0   4.2 

The mean_ppd is the sample average posterior predictive
distribution of the outcome variable (for details see
help('summary.stanreg')).

MCMC diagnostics
              mcse Rhat n_eff
(Intercept)   0.0  1.0  19882
aeromonas     0.0  1.0  12866
cholera       0.0  1.0  12101
eaec_aaic     0.0  1.0  12432
epec_bfpa     0.0  1.0  12231
etec_lt       0.0  1.0  12554
sigma         0.0  1.0  12093
mean_PPD      0.0  1.0  18294
log-posterior 0.0  1.0   6736

For each parameter, mcse is Monte Carlo standard error, n_eff is
a crude measure of effective sample size, and Rhat is the
potential scale reduction factor on split chains (at convergence
Rhat=1).
```

```r
mcmc_trace(y_fits$epec_eae)
```

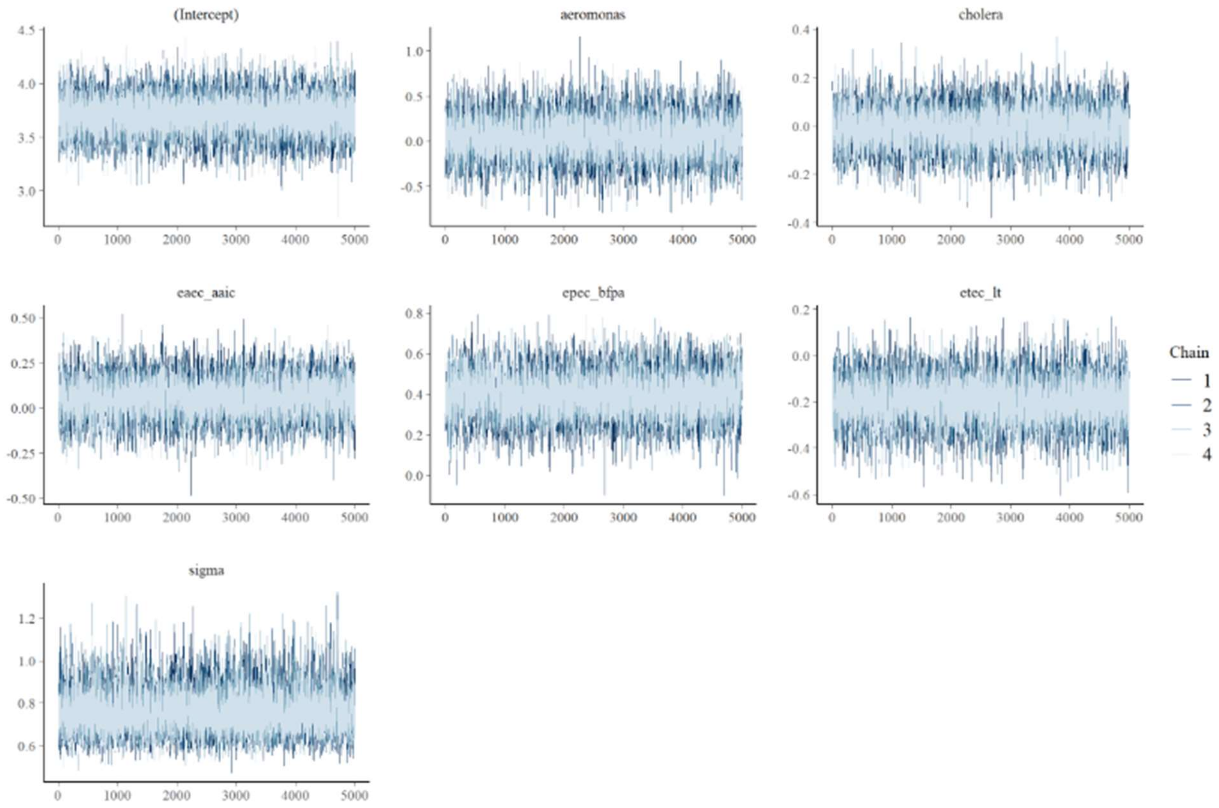

Collate Results

```
# Set number of digits for output
n_digits = 3

# Create tables for reporting results, one for presence/absence,
# the other for log_10 concentration
results =
  list(z = tibble(Covariate = c("(Intercept)",(n_pos %>% sort() %>%
names())[-c(1,length(path_names))])),
       y = tibble(Covariate = c("(Intercept)",(n_pos %>% sort() %>%
names())[-c(1,length(path_names))]))
  )
for(j in (n_pos %>% sort() %>% names())){
  results$z[[j]] = results$y[[j]] = ""

  pmedian =
    z_fits[[j]] %>%
    as.matrix() %>%
    apply(2,median) %>%
    round(n_digits)
  ci =
    posterior_interval(z_fits[[j]],prob = 0.95) %>%
    round(n_digits)
```

```
for(k in 1:length(pmedian)){
    results$z[[j]][ match(gsub("_binary","",names(pmedian))[k],
                          results$z$Covariate) ] =
      paste(pmedian[k],
            " (",
            ci[k,1],
            ", ",
            ci[k,2],
            ")",
            collapse = "",
            sep = "")
  }

  pmedian =
    y_fits[[j]] %>%
    as.matrix() %>%
    apply(2,median) %>%
    round(n_digits)
  pmedian = pmedian[-length(pmedian)]
  ci =
    posterior_interval(z_fits[[j]],prob = 0.95) %>%
    round(n_digits)
  for(k in 1:length(pmedian)){
    results$y[[j]][ match(names(pmedian)[k],
                          results$y$Covariate) ] =
      paste(pmedian[k],
            " (",
            ci[k,1],
            ", ",
            ci[k,2],
            ")",
            collapse = "",
            sep = "")
  }

}
```

<u>Presence/absence</u>

```
results$z %>%
  flextable()
```

Covariate	eaec_aata	aeromonas	cholera	eaec_aaic	epec_bfpa	etec_lt	epec_eae
(Intercept)	-3.51 (-4.967, -2.477)	-2.654 (-3.633, -1.886)	-2.31 (-3.186, -1.605)	-1.765 (-2.482, -1.152)	-2.305 (-3.279, -1.526)	-1.191 (-1.838, -0.601)	-0.656 (-1.298, -0.055)
aeromonas			0.827 (0.115, 1.648)	-0.437 (-1.97, 0.454)	-0.641 (-2.851, 0.63)	0.158 (-0.629, 0.913)	-0.099 (-1.029, 0.715)
cholera				0.227 (-0.106, 0.538)	-0.194 (-0.769, 0.233)	0.196 (-0.129, 0.507)	0.336 (0.011, 0.739)
eaec_aaic					1.011 (0.559, 1.536)	-0.516 (-1.164, 0.032)	0.071 (-0.574, 0.708)
epec_bfpa						0.478 (-0.027, 1.029)	1.172 (0.468, 2.281)
etec_lt							-0.038 (-0.478, 0.379)

Log_10 Concentration

```
results$y %>%
  flextable()
```

Covariate	eaec_aata	aeromonas	cholera	eaec_aaic	epec_bfpa	etec_lt	epec_eae
(Intercept)	5.272 (-4.967, -2.477)	2.849 (-3.633, -1.886)	5.526 (-3.186, -1.605)	3.077 (-2.482, -1.152)	2.945 (-3.279, -1.526)	2.797 (-1.838, -0.601)	3.703 (-1.298, -0.055)
aeromonas			0.048 (0.115, 1.648)	1.745 (-1.97, 0.454)	0.784 (-2.851, 0.63)	0.165 (-0.629, 0.913)	0.051 (-1.029, 0.715)
cholera				0.018 (-0.106, 0.538)	-0.186 (-0.769, 0.233)	-0.031 (-0.129, 0.507)	-0.01 (0.011, 0.739)
eaec_aaic					0.188 (0.559, 1.536)	0.023 (-1.164, 0.032)	0.058 (-0.574, 0.708)
epec_bfpa						0.186 (-0.027, 1.029)	0.392 (0.468, 2.281)
etec_lt							-0.191 (-0.478, 0.379)

Assess Model Fit

Model fit is assessed using the Bayesian p-value based on the following goodness of fit statistic:

$$\sum_{i=1}^{N}\sum_{j=1}^{J}\frac{(y_{ij}-\mathbb{E}(y_{ij})^2)}{Var(y_{ij})}$$

For those unfamiliar with Bayesian p-values, this is a well-established technique to assess model fit in the Bayesian framework. Values close to 0.5 indicate the model fits the data well, while values close to 0 or to 1 indicate a poor fit.

Note that with the sequential GLM modeling approach described above, we have

$$z_{ij} \sim Bernoulli(p_{ij})$$

$$y_{ij} \sim \begin{cases} \delta_0(y) & if\ z_{ij}=0 \\ N(\mu_{ij},\sigma_j^2) & if\ z_{ij}=1 \end{cases}$$

$$where\ logit(p_{ij}) = \alpha_{j0} + \sum_{k<j} y_{ik}\alpha_{jk}\ ,$$

$$\mu_{ij} = \beta_{j0} + \sum_{k<j} y_{ik}\beta_{jk}.$$

$$Hence\ \mathbb{E}(y_{ij}) = \mathbb{E}\left(\mathbb{E}(y_{ij}|z_{ij})\right)$$
$$= \mathbb{E}(\mu_{ij}z_{ij})$$

$$= \mu_{ij} p_{ij}$$
$$Var(y_{ij}) = \mathbb{E}\left(Var(y_{ij}|z_{ij})\right) + Var\left(\mathbb{E}(y_{ij}|z_{ij})\right)$$
$$= \mathbb{E}(\sigma_j{}^2 z_{ij}) + Var\left(\mathbb{E}(y_{ij}|z_{ij})\right)$$
$$= \sigma_j{}^2 p_{ij} + \mu_{ij}{}^2 p_{ij}(1 - p_{ij}).$$

First, get posterior samples of the model parameters:

```
z_samples =
  z_fits %>%
  lapply(as.matrix)
y_samples =
  y_fits %>%
  lapply(as.matrix)
```

Now we need to get samples from the posterior predictive density. This must be done sequentially according to how we fit the models:

```
set.seed(2024)
z_predictions = y_predictions = list()
## eaec_aata
z_predictions$eaec_aata =
  z_fits$eaec_aata %>%
  posterior_predict()
y_predictions$eaec_aata =
  y_fits$eaec_aata %>%
  posterior_predict(newdata = data.frame(whatever =
rep(1,nrow(soil))))
y_predictions$eaec_aata =
  y_predictions$eaec_aata * z_predictions$eaec_aata
## aeromonas
z_predictions$aeromonas =
  z_fits$aeromonas %>%
  posterior_predict()
y_predictions$aeromonas =
  y_fits$aeromonas %>%
  posterior_predict(newdata = data.frame(whatever =
rep(1,nrow(soil))))
y_predictions$aeromonas =
  y_predictions$aeromonas * z_predictions$aeromonas
## cholera
z_predictions$cholera =
  z_samples$cholera[,"(Intercept)"] %x% matrix(1,1,nrow(soil)) +
  z_samples$cholera[,"aeromonas"] * y_predictions$aeromonas #first
term will be repeated correctly
```

```
z_predictions$cholera =
  1.0 * (matrix(runif(prod(dim(z_predictions$cholera))),
                 nrow(z_predictions$cholera),
                 ncol(z_predictions$cholera)) <
           (1.0 / (1.0 + exp(-z_predictions$cholera) ) ) )
y_predictions$cholera =
  y_samples$cholera[,"(Intercept)"] %x% matrix(1,1,nrow(soil)) +
  y_samples$cholera[,"aeromonas"] * y_predictions$aeromonas +
  matrix(rnorm(nrow(y_samples$cholera) * nrow(soil),
                sd = y_samples$cholera[,"sigma"]),
         nrow(y_samples$eaec_aata),
         nrow(soil))
y_predictions$cholera =
  y_predictions$cholera * z_predictions$cholera
## eaec_aaic
z_predictions$eaec_aaic =
  z_samples$eaec_aaic[,"(Intercept)"] %x% matrix(1,1,nrow(soil)) +
  z_samples$eaec_aaic[,"aeromonas"] * y_predictions$aeromonas  +
  z_samples$eaec_aaic[,"cholera"] * y_predictions$cholera
z_predictions$eaec_aaic =
  1.0 * (matrix(runif(prod(dim(z_predictions$eaec_aaic))),
                 nrow(z_predictions$eaec_aaic),
                 ncol(z_predictions$eaec_aaic)) <
           1.0 / (1.0 + exp(-z_predictions$eaec_aaic)))
y_predictions$eaec_aaic =
  y_samples$eaec_aaic[,"(Intercept)"] %x% matrix(1,1,nrow(soil)) +
  y_samples$eaec_aaic[,"aeromonas"] * y_predictions$aeromonas +
  y_samples$eaec_aaic[,"cholera"] * y_predictions$cholera +
  matrix(rnorm(nrow(y_samples$eaec_aaic) * nrow(soil),
                sd = y_samples$eaec_aaic[,"sigma"]),
         nrow(y_samples$eaec_aata),
         nrow(soil))
y_predictions$eaec_aaic =
  y_predictions$eaec_aaic * z_predictions$eaec_aaic
## epec_bfpa
z_predictions$epec_bfpa =
  z_samples$epec_bfpa[,"(Intercept)"] %x% matrix(1,1,nrow(soil)) +
  z_samples$epec_bfpa[,"aeromonas"] * y_predictions$aeromonas  +
  z_samples$epec_bfpa[,"cholera"] * y_predictions$cholera +
  z_samples$epec_bfpa[,"eaec_aaic"] * y_predictions$eaec_aaic
z_predictions$epec_bfpa =
  1.0 * (matrix(runif(prod(dim(z_predictions$epec_bfpa))),
                 nrow(z_predictions$epec_bfpa),
                 ncol(z_predictions$epec_bfpa)) <
           1.0  / (1.0 + exp(-z_predictions$epec_bfpa)))
```

```
y_predictions$epec_bfpa =
  y_samples$epec_bfpa[,"(Intercept)"] %x% matrix(1,1,nrow(soil)) +
  y_samples$epec_bfpa[,"aeromonas"] * y_predictions$aeromonas +
  y_samples$epec_bfpa[,"cholera"] * y_predictions$cholera +
  y_samples$epec_bfpa[,"eaec_aaic"] * y_predictions$eaec_aaic +
  matrix(rnorm(nrow(y_samples$epec_bfpa) * nrow(soil),
               sd = y_samples$epec_bfpa[,"sigma"]),
         nrow(y_samples$eaec_aata),
         nrow(soil))
y_predictions$epec_bfpa =
  y_predictions$epec_bfpa * z_predictions$epec_bfpa
## etec_lt
z_predictions$etec_lt =
  z_samples$etec_lt[,"(Intercept)"] %x% matrix(1,1,nrow(soil)) +
  z_samples$etec_lt[,"aeromonas"] * y_predictions$aeromonas  +
  z_samples$etec_lt[,"cholera"] * y_predictions$cholera +
  z_samples$etec_lt[,"eaec_aaic"] * y_predictions$eaec_aaic +
  z_samples$etec_lt[,"epec_bfpa"] * y_predictions$epec_bfpa
z_predictions$etec_lt =
  1.0 * (matrix(runif(prod(dim(z_predictions$etec_lt))),
                nrow(z_predictions$etec_lt),
                ncol(z_predictions$etec_lt)) <
           1.0 / (1.0 + exp(-z_predictions$etec_lt)))
y_predictions$etec_lt =
  y_samples$etec_lt[,"(Intercept)"] %x% matrix(1,1,nrow(soil)) +
  y_samples$etec_lt[,"aeromonas"] * y_predictions$aeromonas +
  y_samples$etec_lt[,"cholera"] * y_predictions$cholera +
  y_samples$etec_lt[,"eaec_aaic"] * y_predictions$eaec_aaic +
  y_samples$etec_lt[,"epec_bfpa"] * y_predictions$epec_bfpa +
  matrix(rnorm(nrow(y_samples$etec_lt) * nrow(soil),
               sd = y_samples$etec_lt[,"sigma"]),
         nrow(y_samples$eaec_aata),
         nrow(soil))
y_predictions$etec_lt =
  y_predictions$etec_lt * z_predictions$etec_lt
## epec_eae
z_predictions$epec_eae =
  z_samples$epec_eae[,"(Intercept)"] %x% matrix(1,1,nrow(soil)) +
  z_samples$epec_eae[,"aeromonas"] * y_predictions$aeromonas  +
  z_samples$epec_eae[,"cholera"] * y_predictions$cholera +
  z_samples$epec_eae[,"eaec_aaic"] * y_predictions$eaec_aaic +
  z_samples$epec_eae[,"epec_bfpa"] * y_predictions$epec_bfpa +
  z_samples$epec_eae[,"etec_lt"] * y_predictions$etec_lt
z_predictions$epec_eae =
  1.0 * (matrix(runif(prod(dim(z_predictions$epec_eae))),
                nrow(z_predictions$epec_eae),
                ncol(z_predictions$epec_eae)) <
           1.0 / (1.0 + exp(-z_predictions$epec_eae)))
```

```
y_predictions$epec_eae =
  y_samples$epec_eae[,"(Intercept)"] %x% matrix(1,1,nrow(soil)) +
  y_samples$epec_eae[,"aeromonas"] * y_predictions$aeromonas +
  y_samples$epec_eae[,"cholera"] * y_predictions$cholera +
  y_samples$epec_eae[,"eaec_aaic"] * y_predictions$eaec_aaic +
  y_samples$epec_eae[,"epec_bfpa"] * y_predictions$epec_bfpa +
  y_samples$epec_eae[,"etec_lt"] * y_predictions$etec_lt +
  matrix(rnorm(nrow(y_samples$epec_eae) * nrow(soil),
               sd = y_samples$epec_eae[,"sigma"]),
         nrow(y_samples$eaec_aata),
         nrow(soil))
y_predictions$epec_eae =
  y_predictions$epec_eae * z_predictions$epec_eae
```

Below is the function to obtain the GOF statistics (one for the observed data and one for the predictive sample) for a given posterior draw.

```
gof_stat = function(iter){
  dt.soil_sim =
    soil %>%
    dplyr::select(all_of(names(z_fits)))

  for(j in names(z_fits)) dt.soil_sim[[j]] =
y_predictions[[j]][iter,]
  dt.soil_sim %<>%
    bind_cols(dt.soil_sim %>%
                mutate_all(function(x) ifelse(is.na(x),0, 1)) %>%
                rename_all(function(x) paste0(x,"_binary")))

  PrGr0 =
    mu =
    Var =
    Exp =
    matrix(0.0,nrow(dt.soil_sim),NROW(n_pos))

  ## Compute GOF stat for simulated data
  for(j in 1:NROW(n_pos)){
    mm = model.matrix(formula(z_fits[[j]]), # Formula is the same
for z_fits and y_fits
                      data = dt.soil_sim)
    PrGr0[,j] =
      1.0 / drop(1.0 + exp(-mm %*% z_samples[[j]][iter,]))

    mu[,j] = mm %*% y_samples[[j]][iter,-ncol(y_samples[[j]])]

    Exp[,j] = PrGr0[,j] * mu[,j]
```

```
Var[,j] =
      PrGr0[,j] * y_samples[[j]][iter,ncol(y_samples[[j]])]^2 +
      PrGr0[,j] * (1.0 - PrGr0[,j]) * mu[,j]^2
  }

  GOF_sim =
    sum( (as.matrix(dt.soil_sim %>%
                      dplyr::select(all_of(names(z_fits)))) -
Exp)^2 / Var )

  ## Compute GOF stat for observed data
  for(j in 1:NROW(n_pos)){
    mm = model.matrix(formula(z_fits[[j]]),
                      data = soil)
    PrGr0[,j] =
      1.0 / drop(1.0 + exp(-mm %*% z_samples[[j]][iter,]))

    mu[,j] = mm %*% y_samples[[j]][iter,-ncol(y_samples[[j]])]

    Exp[,j] = PrGr0[,j] * mu[,j]

    Var[,j] =
      PrGr0[,j] * y_samples[[j]][iter,ncol(y_samples[[j]])]^2 +
      PrGr0[,j] * (1.0 - PrGr0[,j]) * mu[,j]^2
  }

  GOF_obs =
    sum( (as.matrix(soil %>%
                      dplyr::select(all_of(names(z_fits)))) -
Exp)^2 / Var )

  return(c(GOF_sim = GOF_sim,
           GOF_obs = GOF_obs))
}
```

Now to compute and plot.

```
GOF_stats =
  sapply(1:nrow(z_samples[[1]]),gof_stat) %>%
  t() %>%
  as_tibble()
```

```r
plot(GOF_stats,
     pch = 16,
     col = c("indianred","steelblue")[1L + (GOF_stats$GOF_sim <
GOF_stats$GOF_obs)],
     bty = 'l',
     xlim = quantile(GOF_stats$GOF_sim,c(0.01,0.99)),
     ylim = quantile(GOF_stats$GOF_obs,c(0.01,0.99)))
legend('topright',
       bty = 'n',
       cex = 2,
       legend = paste0("Bayesian p-value =
",round(mean(GOF_stats$GOF_sim < GOF_stats$GOF_obs),2)))
```

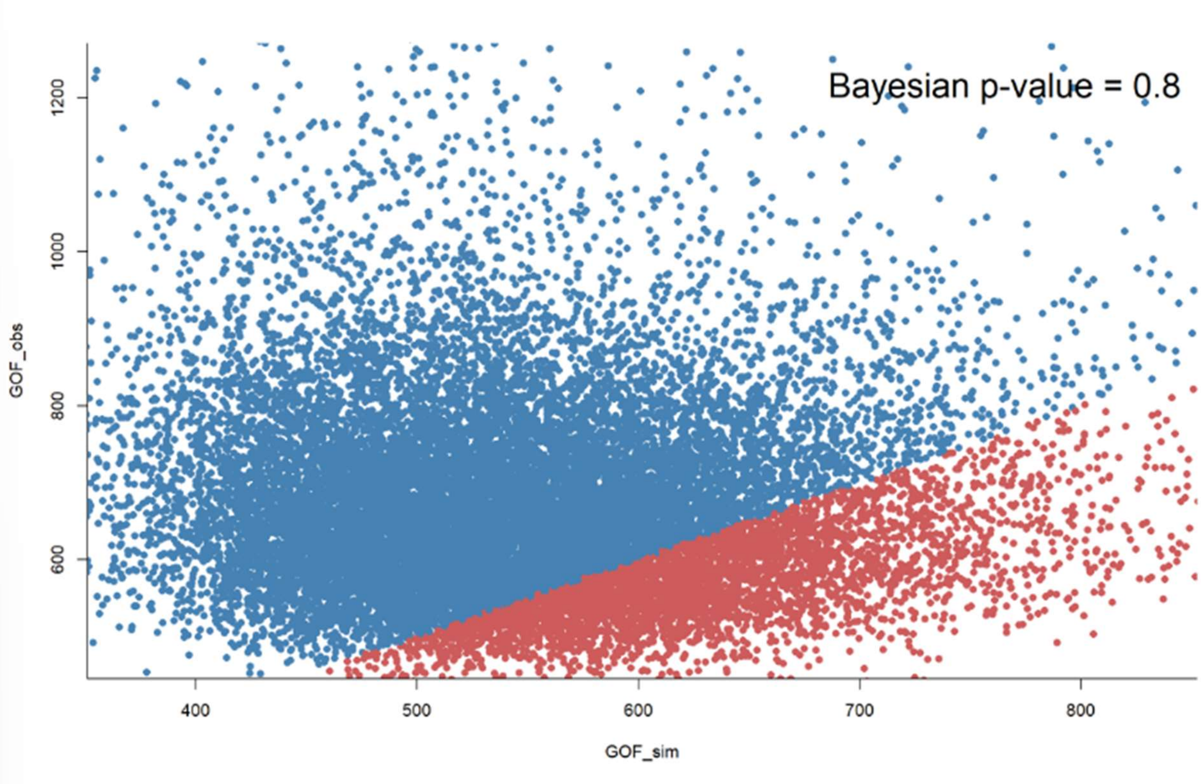

Behavioral Modeling

Model Fitting

We consider four different models: Poisson, negative binomial, zero-inflated Poisson (ZIP), and zero-inflated negative binomial (ZINB). We then use Bayes factors to determine the best fitting model.

Set up:

```
beh_names = names(behavior)[-c(1:3)]

b_fits = list()
for(j in beh_names) b_fits[[j]] = list()
```

For each model, we need to compile the STAN code. After that, we can simply change the data being fed into that STAN model and run the HMC algorithm.

<u>Poisson</u>

```
b_fits$hand_to_object$poisson =
  brm(y ~ offset(log(time_min)) + (1|age),
      family = "poisson",
      data =
        behavior %>%
        rename(y = hand_to_object),
      save_pars = save_pars(all = TRUE),
      iter = 10000,
      seed = 2024,
      cores = 4)
for(j in beh_names[-1]){
  b_fits[[j]]$poisson =
    b_fits$hand_to_object$poisson %>%
    update(newdata =
             behavior %>%
             rename(y = !!j),
           seed = 2024,
           cores = 4)
}
```

Check for convergence:

```
lapply(b_fits,
       function(X) rhat(X$poisson)) %>%
  unlist() %>%
  max()
```

```
[1] 1.012148
```

Negative binomial

```
b_fits$hand_to_object$negbinomial =
  brm(y ~ offset(log(time_min)) + (1|age),
      family = "negbinomial",
      data =
        behavior %>%
        rename(y = hand_to_object),
      save_pars = save_pars(all = TRUE),
      iter = 10000,
      seed = 2024,
      cores = 4)
for(j in beh_names[-1]){
  b_fits[[j]]$negbinomial =
    b_fits$hand_to_object$negbinomial %>%
    update(newdata =
             behavior %>%
             rename(y = !!j),
           seed = 2024,
           cores = 4)
}
```

Check for convergence:

```
lapply(b_fits,
       function(X) rhat(X$negbinomial)) %>%
  unlist() %>%
  max()
```

```
[1] 1.009607
```

Zero-inflated Poisson

```
b_fits$hand_to_object$zip =
  brm(y ~ offset(log(time_min)) + (1|age),
      family = "zero_inflated_poisson",
      data =
        behavior %>%
        rename(y = hand_to_object),
      save_pars = save_pars(all = TRUE),
      iter = 10000,
      seed = 2024,
      cores = 4)
for(j in beh_names[-1]){
  b_fits[[j]]$zip =
    b_fits$hand_to_object$zip %>%
    update(newdata =
             behavior %>%
             rename(y = !!j),
           seed = 2024,
           cores = 4)
}
```

Check for convergence:

```
lapply(b_fits,
       function(X) rhat(X$zip)) %>%
  unlist() %>%
  max()
```

```
[1] 1.058392
```

Zero-inflated Negative Binomial

```
b_fits$hand_to_object$zinb =
  brm(y ~ offset(log(time_min)) + (1|age),
      family = "zero_inflated_negbinomial",
      data =
        behavior %>%
        rename(y = hand_to_object),
      save_pars = save_pars(all = TRUE),
      iter = 10000,
      seed = 2024,
      cores = 4)
for(j in beh_names[-1]){
  b_fits[[j]]$zinb =
    b_fits$hand_to_object$zinb %>%
    update(newdata =
             behavior %>%
             rename(y = !!j),
           seed = 2024,
           cores = 4)
}
```

Check for convergence:

```
lapply(b_fits,
       function(X) rhat(X$zinb)) %>%
  unlist() %>%
  max()
```

```
[1] 1.009326
```

Model Selection

We select the best model for each behavior via Bayes factors.

```
bf_by_behavior = list()
for(j in names(b_fits)){
  cat(paste0("\n--- ",j))
  bf_by_behavior[[j]] =
    matrix(0.0,4,4,
           dimnames = list(c("poisson","negbinomial","zip","zinb"),

c("poisson","negbinomial","zip","zinb")))
  bf_by_behavior[[j]][lower.tri(bf_by_behavior[[j]])] = NA
  diag(bf_by_behavior[[j]]) = NA
  bf_by_behavior[[j]][1,2] =
    bayes_factor(b_fits[[j]]$poisson,
                 b_fits[[j]]$negbinomial)$bf
  bf_by_behavior[[j]][1,3] =
    bayes_factor(b_fits[[j]]$poisson,
                 b_fits[[j]]$zip)$bf
  bf_by_behavior[[j]][1,4] =
    bayes_factor(b_fits[[j]]$poisson,
                 b_fits[[j]]$zinb)$bf
  bf_by_behavior[[j]][2,3] =
    bayes_factor(b_fits[[j]]$negbinomial,
                 b_fits[[j]]$zip)$bf
  bf_by_behavior[[j]][2,4] =
    bayes_factor(b_fits[[j]]$negbinomial,
                 b_fits[[j]]$zinb)$bf
  bf_by_behavior[[j]][3,4] =
    bayes_factor(b_fits[[j]]$zip,
                 b_fits[[j]]$zinb)$bf
}
```

```
best_fits = list()

for(j in names(bf_by_behavior)){
  bf_by_behavior[[j]][lower.tri(bf_by_behavior[[j]])] =
    1.0 / t(bf_by_behavior[[j]])[lower.tri(bf_by_behavior[[j]])]
  diag(bf_by_behavior[[j]]) = 1.0

  best_model_name =
    rownames(bf_by_behavior[[j]])[which(apply(bf_by_behavior[[j]],
                                              1,
                                              function(x) all(x >=
1.0)))]
  best_fits[[j]] =
    b_fits[[j]][[best_model_name]]
}
```

Below are the estimated parameters of the behavioral distributions. Note that for the zero probabilities for the ZIP and ZINB, the results are on the scale of the linear predictor, i.e., the untransformed regression coefficients. This is because the log(minutes observed) was used as a

covariate rather than an offset for this component. ("B" represents baby or infant, "T" represents toddler, and "C" represents child.)

```
best_fit_summary =
  tibble(Behavior = rep(gsub("_","-",names(best_fits)),
                              each = 3),
         Age = rep(c("B","C","T"), length(best_fits)),
         `Poi mean` = NA,
         `NB mean` = NA,
         `NB dispersion` = NA,
         `ZIP Poi mean` = NA,
         `ZIP zero prob Intercept` = NA,
         `ZIP zero prob logtime (min)` = NA,
         `ZINB NB mean` = NA,
         `ZINB NB dispersion` = NA,
         `ZINB zero prob Intercept` = NA,
         `ZINB zero prob logtime (min)` = NA)
for(i in 1:length(best_fits)){
  if(best_fits[[i]]$family$family == "poisson"){
    best_fit_summary$`Poi mean`[3 * (i - 1) + 1:3] =
      exp(coef(best_fits[[i]])$age[,"Estimate","Intercept"])
  }
  if(best_fits[[i]]$family$family == "negbinomial"){
    best_fit_summary$`NB mean`[3 * (i - 1) + 1:3] =
      exp(coef(best_fits[[i]])$age[,"Estimate","Intercept"])
    best_fit_summary$`NB dispersion`[3 * (i - 1) + 1:3] =
      summary(best_fits[[i]])$spec_pars$Estimate
  }
  if(best_fits[[i]]$family$family == "zero_inflated_poisson"){
    best_fit_summary$`ZIP Poi mean`[3 * (i - 1) + 1:3] =
      exp(coef(best_fits[[i]])$age[,"Estimate","Intercept"])
    best_fit_summary$`ZIP zero prob Intercept`[3 * (i - 1) + 1:3] =
      coef(best_fits[[i]])$age[,"Estimate","zi_Intercept"]
    best_fit_summary$`ZIP zero prob logtime (min)`[3 * (i - 1) +
1:3] =
      coef(best_fits[[i]])$age[,"Estimate","zi_logtime_min"]
  }
if(best_fits[[i]]$family$family == "zero_inflated_negbinomial"){
    best_fit_summary$`ZINB NB mean`[3 * (i - 1) + 1:3] =
      exp(coef(best_fits[[i]])$age[,"Estimate","Intercept"])
    best_fit_summary$`ZINB NB dispersion`[3 * (i - 1) + 1:3] =
      summary(best_fits[[i]])$spec_pars$Estimate
    best_fit_summary$`ZINB zero prob Intercept`[3 * (i - 1) + 1:3]
=
      coef(best_fits[[i]])$age[,"Estimate","zi_Intercept"]
    best_fit_summary$`ZINB zero prob logtime (min)`[3 * (i - 1) +
1:3] =
      coef(best_fits[[i]])$age[,"Estimate","zi_logtime_min"]
  }
}
```

```
best_fit_summary %>%
  flextable() %>%
  merge_v(1) %>%
  hline(1:3*3) %>%
  colformat_double(digits = 3)
```

Behavior	Age	Poi mean	NB mean	NB dispersion	ZIP Poi mean	ZIP zero prob Intercept	ZIP zero prob logtime (min)	ZINB NB mean	ZINB NB dispersion	ZINB zero prob Intercept	ZINB zero prob logtime (min)
hand-to-object	B		0.367	2.338							
	C		0.443	2.338							
	T		0.361	2.338							
hand-to-soil	B		0.187	0.854							
	C		0.207	0.854							
	T		0.186	0.854							
mouth-to-object	B		0.062	0.319							
	C		0.027	0.319							
	T		0.042	0.319							
geophagy	B				0.013	0.014	-1.523				
	C				0.002	1.164	-1.523				
	T				0.004	0.129	-1.523				
mouth-to-hand	B		0.323	1.214							
	C		0.161	1.214							
	T		0.209	1.214							

Monte Carlo Simulation

First, we will draw new behavior counts in a 1hr period through the posterior predictive distributions.

```
set.seed(2024)
behavior_draws = list()

for(j in names(best_fits)){
  behavior_draws[[j]] =
    best_fits[[j]] %>%
    posterior_predict(newdata = tibble(age = c("B","T","C"),
                                       time_min = 60))
  colnames(behavior_draws[[j]]) = c("B","T","C")
}
```

```
behavior_draws_compiled = behavior_draws
for(j in names(behavior_draws_compiled)){
  behavior_draws_compiled[[j]] %<>%
    as_tibble() %>%
    mutate(Behavior = j) %>%
    relocate(Behavior)
}
behavior_draws_compiled =
  do.call(bind_rows,
          behavior_draws_compiled) %>%
  pivot_longer(cols = B:C,
               names_to = "Age",
               values_to = "Count")

behavior_draws_compiled %>%
  ggplot(aes(y = Count,
             x = Behavior,
             fill = Age)) +
  geom_boxplot(outliers = FALSE) +
  ylab("Count / hr") +
  scale_fill_viridis_d() +
    theme(
      panel.background = element_rect(fill = "white"),
      panel.grid.major = element_line(color = "white"),
      panel.grid.minor = element_blank(),
      axis.line = element_line(color = "black"),
      axis.text.y = element_text(size = 16),
      axis.text.x = element_text(size = 16,angle = -45, hjust = 0),
      axis.title = element_text(size = 18, face = "bold"),
      axis.ticks = element_line(color = "black"),
      legend.background = element_rect(fill = "white"),
      legend.key = element_blank(),
      legend.text = element_text(size = 16),
      legend.title = element_text(size = 18, face = "bold")
      )
```

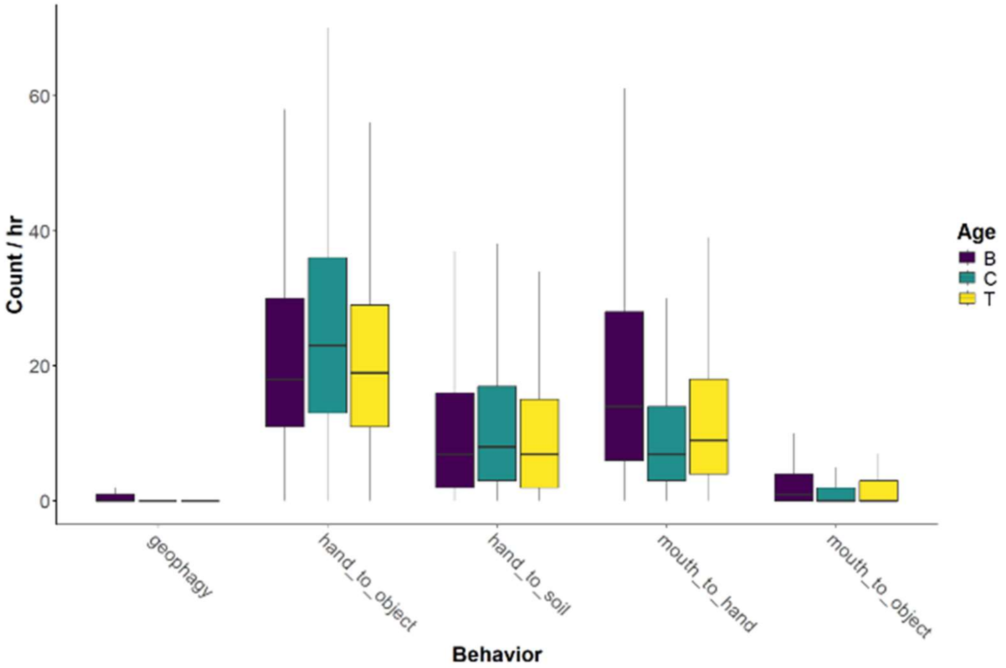

Next we will draw values/fix constants from the extant literature.

```
n_sim = nrow(behavior_draws$mouth_to_hand)
set.seed(2024)

## Helper function to get mean and sd from empirical quantiles
find_mean_and_sd = function(probs,
                            obs_quantiles,
                            init_for_optim){

  objective_function = function(x){
    sum( (obs_quantiles - qnorm(probs,
                                mean = x[1],
                                sd = exp(x[2])))^2 )
  }

  optim(init_for_optim,
        objective_function)
}

## Transfer Efficiencies
trans_soil_hand =
  rlnorm(n_sim, meanlog = 0.11, sdlog = 2.0) #Ozkaynak et al
trans_object_hand =
  rtrunc(n_sim, "norm", a = 0, b =1, mean = 0.23, sd=0.22) # Julian,
et al
trans_hand_mouth =
  rbeta(n_sim,shape1 = 5.2, shape2 = 2.6) # Kwong et al
trans_object_mouth =
  rbeta(n_sim, shape1 = 2, shape2 = 8)  # Ozkaynak et al
```

```r
# Surface areas
child_hand_surf_area_cm2 = list()
child_hand_surf_area_cm2$B =
  rtri(n_sim, min = 74.59, max = 119.18, mode=98.31) #Kwong et al
2019
child_hand_surf_area_cm2$T =
  rtri(n_sim, min = 80.01, max = 177.38, mode=121.18) #Kwong et al
2019
child_hand_surf_area_cm2$C =
  rtri(n_sim, min = 139.63, max = 153.28, mode=146.47) #Kwong et al
2019
object_surf_area_cm2 =
  rexp(n_sim,rate=0.11) #Kwong et al 2019

# Other miscellaneous constants
soil_handful_g =
  0.001 * rbeta(n_sim, shape1 = 5.3, shape2=158.6) #Kwong, et al
2019

hand_loading_g_cm2 =
  0.001 * rtri(n_sim, min = 0, max = 0.95, mode=0.1) #Kwong et al
2019

obj_loading_g_cm2 =
  0.001 * rlnorm(n_sim, meanlog = 0.11, sdlog = 2.0) #Ozkaynak et
al # Treating objects like hands

temp =
  find_mean_and_sd(c(5, 25, 50, 75, 95, 99)/100,
                   c(0.10,0.16,0.21,0.25,0.31,0.60),
                   c(0.21,0.05))
temp$convergence == 0
```

```
[1] TRUE
```

```r
print(c(mean = temp$par[1],sd = exp(temp$par[2])))
```

```
mean        sd
0.2299022   0.1080376
```

```r
child_frac_hand_touch_soil =
  rtrunc(n_sim,
         "norm",
         a = 0, b =1,
         mean = temp$par[1],
         sd = exp(temp$par[2])) #Auyueng et al, 2008

temp =
  find_mean_and_sd(c(5, 25, 50, 75, 95, 99)/100,
                   c(0.12,0.14,0.16,0.19,0.30,0.42),
                   c(0.16,log(0.05)))
temp$convergence == 0
```

```
[1] TRUE
```

```r
print(c(mean = temp$par[1],sd = exp(temp$par[2])))
```

```
mean            sd
0.1933396       0.0730457
```

```r
child_frac_hand_touch_mouth =
  rtrunc(n_sim,
         "norm",
         a = 0, b =1,
         mean = temp$par[1],
         sd = exp(temp$par[2])) #Auyueng et al, 2008

temp =
  find_mean_and_sd(c(5, 25, 50, 75, 95, 99)/100,
                   c(0.13,0.14,0.15,0.17,0.20,0.23),
                   c(0.15,log(0.05)))
temp$convergence == 0
```

```
[1] TRUE
```

```r
print(c(mean = temp$par[1],sd = exp(temp$par[2])))
```

```
mean            sd
0.16015715      0.02538482
```

```
child_frac_hand_touch_object =
  rtrunc(n_sim,
         "norm",
         a = 0, b =1,
         mean = temp$par[1],
         sd = exp(temp$par[2])) #Auyueng et al, 2008

max_obj_loading_g_cm2 =
  0.001 * runif(n_sim, 6, 8) #Ozkaynak et al # Treating objects like
hands
```

Set up objects for exposure calculations and compute for each MC draw. Note that since soil samples are exchangeable, we can simply use the first column of each y_prediction matrix.

```
exposure_dose_per_hour_culture = exposure_dose_per_hour = list()
for(age in c("B","T","C")){
  exposure_dose_per_hour[[age]] =
  exposure_dose_per_hour_culture[[age]] =
    matrix(0.0, n_sim, length(y_fits),
            dimnames = list(NULL, names(y_fits)))
}

for(age in names(exposure_dose_per_hour)){
  for(j in names(y_fits)){
    exposure_dose_per_hour[[age]][,j] =
      ifelse(y_predictions[[j]][,1] == 0, 0,
10^y_predictions[[j]][,1]) *
       ( (trans_soil_hand *
           child_hand_surf_area_cm2[[age]] *
           child_frac_hand_touch_soil *
           child_frac_hand_touch_mouth *
           trans_hand_mouth *
           hand_loading_g_cm2 *
           sapply(1:n_sim,function(i){
             min(behavior_draws$hand_to_soil[i,age],
                 behavior_draws$mouth_to_hand[i,age])
           })
         ) +
```

```
( trans_object_hand *
              child_hand_surf_area_cm2[[age]] *
              frac_hand_touch_object *
              child_frac_hand_touch_mouth *
              trans_hand_mouth *
              hand_loading_g_cm2 *
              sapply(1:n_sim,function(i){
                min(behavior_draws$hand_to_object[i,age],
                      behavior_draws$mouth_to_hand[i,age])
              })
            ) +
          ( trans_object_mouth *
              object_surf_area_cm2 *
              sapply(1:n_sim,function(i){
                min(obj_loading_g_cm2,
                      max_obj_loading_g_cm2)
                }) *
              behavior_draws$mouth_to_object[,age]
            ) +
          (soil_handful_g *
             behavior_draws$geophagy[,age]))
  }
}
```

Results

Tabular Form

```
exposure_results_table = list()
for(j in names(exposure_dose_per_hour)){
  exposure_results_table[[j]] =
    tibble(Organism =
             names(y_fits),
           `Pr(+)` =
             apply(exposure_dose_per_hour[[j]],2,function(x) mean(x
> 0)),
           `Median | +` =
             apply(exposure_dose_per_hour[[j]],2,function(x)
median(x[which(x > 0)])),
           lower =
             apply(exposure_dose_per_hour[[j]],2,function(x)
quantile(x[which(x > 0)],probs = 0.025)),
           upper =
             apply(exposure_dose_per_hour[[j]],2,function(x)
quantile(x[which(x > 0)],probs = 0.975))) %>%
```

```
mutate(`Median | +` = round(log10(`Median | +`),2),
           `95% PI` = paste("(",
                            round(log10(lower),2),
                            ", ",
                            round(log10(upper),2),
                            ")",
                            sep = ""),.keep = "unused") %>%
    rename_with( ~ paste(switch(j, B = "Baby", T = "Toddler", C =
"Child"),
                         .x, sep = "_"))
}

exposure_results_table %>%
  do.call(what = bind_cols) %>%
  select(-Toddler_Organism,-Child_Organism) %>%
  rename(Organism = Baby_Organism) %>%
  flextable() %>%
  separate_header()
```

	Baby			Toddler			Child		
Organism	Pr(+)	Median | +	95% PI	Pr(+)	Median | +	95% PI	Pr(+)	Median | +	95% PI
eaec_aata	0.03005	2.96	(0.6, 5.02)	0.02945	2.95	(-0.13, 5.05)	0.02885	3.01	(-0.27, 5.01)
aeromonas	0.06715	0.46	(-2.99, 4.08)	0.06620	0.49	(-3.27, 4.11)	0.06565	0.58	(-3.49, 4.2)
cholera	0.11780	3.28	(1.08, 5.36)	0.11605	3.27	(0.72, 5.4)	0.11435	3.34	(0.62, 5.34)
eaec_aaic	0.17330	0.84	(-1.48, 5.84)	0.17070	0.88	(-2.16, 5.84)	0.16880	0.91	(-1.93, 5.58)
epec_bfpa	0.18140	0.97	(-1.96, 4.26)	0.17780	0.97	(-2.22, 4.37)	0.17660	1.04	(-2.33, 4.35)
etec_lt	0.27610	0.69	(-2.03, 3.44)	0.27210	0.71	(-2.24, 3.45)	0.26915	0.75	(-2.34, 3.5)
epec_eae	0.48060	1.68	(-1.22, 4.75)	0.47380	1.69	(-1.38, 4.87)	0.46845	1.73	(-1.54, 4.88)

Graphical Form

```
exposure_results =
  exposure_dose_per_hour$B %>%
  as_tibble() %>%
  mutate(Age = "Baby") %>%
  bind_rows(exposure_dose_per_hour$T %>%
              as_tibble() %>%
              mutate(Age = "Toddler")) %>%
  bind_rows(exposure_dose_per_hour$C %>%
              as_tibble() %>%
              mutate(Age = "Child")) %>%
  pivot_longer(!Age,
               names_to = "Organism",
               values_to = "Concentration") %>%
  mutate(Organism =
           case_match(Organism,
                      "eaec_aata" ~ "EAEC aata",
                      "aeromonas" ~ "Aeromonas",
                      "cholera" ~ "Cholera",
                      "eaec_aaic" ~ "EAEC aaic",
                      "epec_bfpa" ~ "EPEC bfpa",
                      "etec_lt" ~ "ETEC LT",
                      "epec_eae" ~ "EPEC eae"))
```

```
exposure_results %>%
  group_by(Age,Organism) %>%
  summarize_if(is.numeric,
               list(`Pr(+)` =
                      function(x) mean(x > 0))) %>%
  ggplot(aes(y = `Pr(+)`,
             x = Organism,
             fill = Age)) +
  geom_bar(position = 'dodge', stat = 'identity') +
  scale_fill_viridis_d() +
    theme(
      panel.background = element_rect(fill = "white"),
      panel.grid.major = element_line(color = "white"),
      panel.grid.minor = element_blank(),
      axis.line = element_line(color = "black"),
      axis.text.y = element_text(size = 16),
      axis.text.x = element_text(size = 16,angle = -45, hjust = 0),
      axis.title = element_text(size = 18, face = "bold"),
      axis.ticks = element_line(color = "black"),
      legend.background = element_rect(fill = "white"),
      legend.key = element_blank(),
      legend.text = element_text(size = 16),
      legend.title = element_text(size = 18, face = "bold")
      )
```

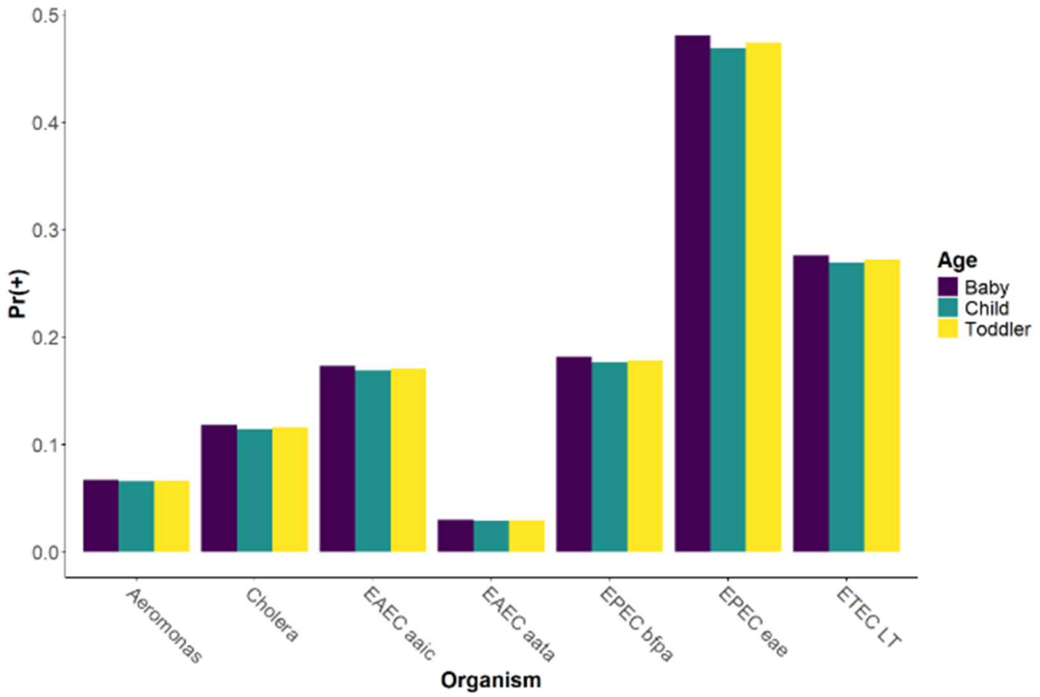


```
IQR_bounds =
  exposure_results %>%
  filter(!near(Concentration, 0)) %>%
  mutate(Concentration = log10(Concentration)) %>%
  group_by(Age,Organism) %>%
  summarize(lower = quantile(Concentration,0.25) - 1.5 *
IQR(Concentration),
            upper = quantile(Concentration,0.75) + 1.5 *
IQR(Concentration))

exposure_results %>%
  filter(!near(Concentration, 0)) %>%
  mutate(Concentration = log10(Concentration)) %>%
  ggplot(aes(y = Concentration,
             x = Organism,
             fill = Age)) +
  geom_violin(bounds = range(c(IQR_bounds$lower,
                              IQR_bounds$upper))) +
  scale_fill_viridis_d() +
  scale_y_continuous(breaks = c(-4:6),
                     labels = 10^c(-4:6)) +
  geom_hline(yintercept = c(-4:6),
             color = "gray30",
             linetype = "dashed",
             alpha = 0.5) +
    theme(
```

```
panel.background = element_rect(fill = "white"),
    panel.grid.major = element_line(color = "white"),
    panel.grid.minor = element_blank(),
    axis.line = element_line(color = "black"),
    axis.text.y = element_text(size = 16),
    axis.text.x = element_text(size = 16,angle = -45, hjust = 0),
    axis.title = element_text(size = 18, face = "bold"),
    axis.ticks = element_line(color = "black"),
    legend.background = element_rect(fill = "white"),
    legend.key = element_blank(),
    legend.text = element_text(size = 16),
    legend.title = element_text(size = 18, face = "bold")
    )
```

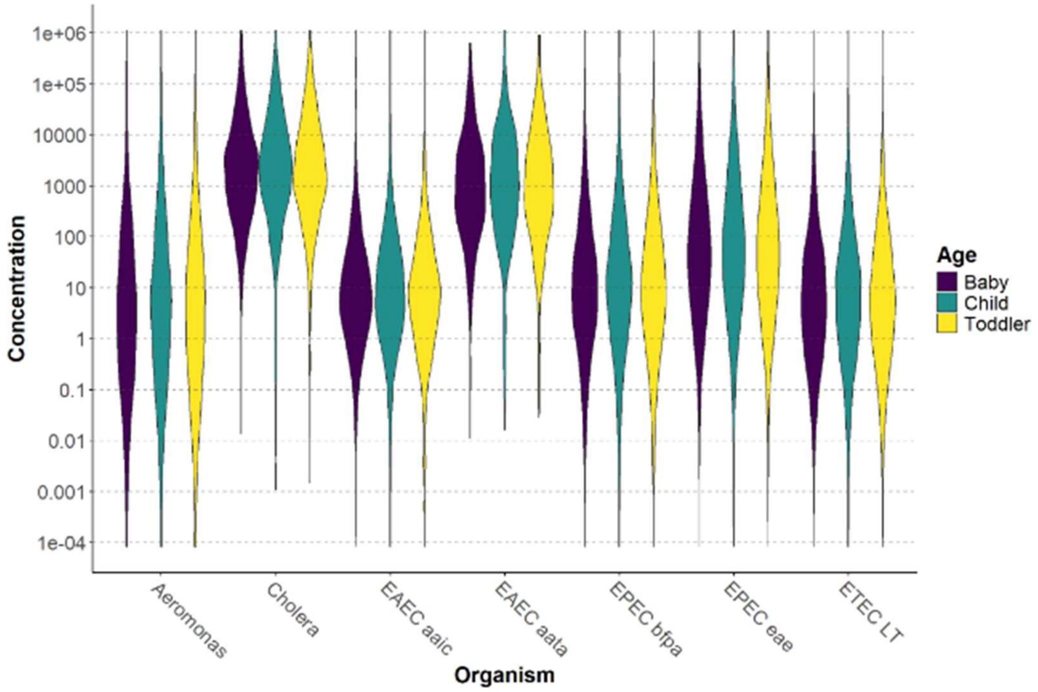

Multipathogen Exposure

Graphically display the probability of multipathogen exposure:

```
multipath_exposure = list()
for(j in names(exposure_dose_per_hour)){
  multipath_exposure[[j]] =
    tibble(Age = switch(j, B = "Baby", T = "Toddler", C = "Child"),
           `N pathogens` =
             apply(exposure_dose_per_hour[[j]],1,function(x)
sum(x>0))
    )
}
multipath_exposure %<>%
  bind_rows() %>%
  mutate(`N pathogens` = ifelse(`N pathogens` >= 4,
"4+",as.character(`N pathogens`))) %$%
  table(Age, `N pathogens`) %>%
  prop.table(1) %>%
  as_tibble() %>%
  rename(`Posterior prob` = n) %>%
  mutate(`N pathogens` = factor(`N pathogens`,
                                levels =
rev(c("0","1","2","3","4+"))))
```

```
multipath_exposure %>%
  mutate(Age = factor(Age, levels = c("Baby","Toddler","Child")))
%>%
  ggplot(aes(x = `Posterior prob`,
             y = Age,
             fill = `N pathogens`)) +
  geom_bar(stat = "identity",position = "stack") +
      theme(
      panel.background = element_rect(fill = "white"),
      panel.grid.major = element_line(color = "white"),
      panel.grid.minor = element_blank(),
      axis.line = element_line(color = "black"),
      axis.text = element_text(size = 16),
      axis.title = element_text(size = 18, face = "bold"),
      axis.ticks = element_line(color = "black"),
      legend.background = element_rect(fill = "white"),
      legend.key = element_blank(),
      legend.text = element_text(size = 16),
      legend.title = element_text(size = 18, face = "bold")
      )
```

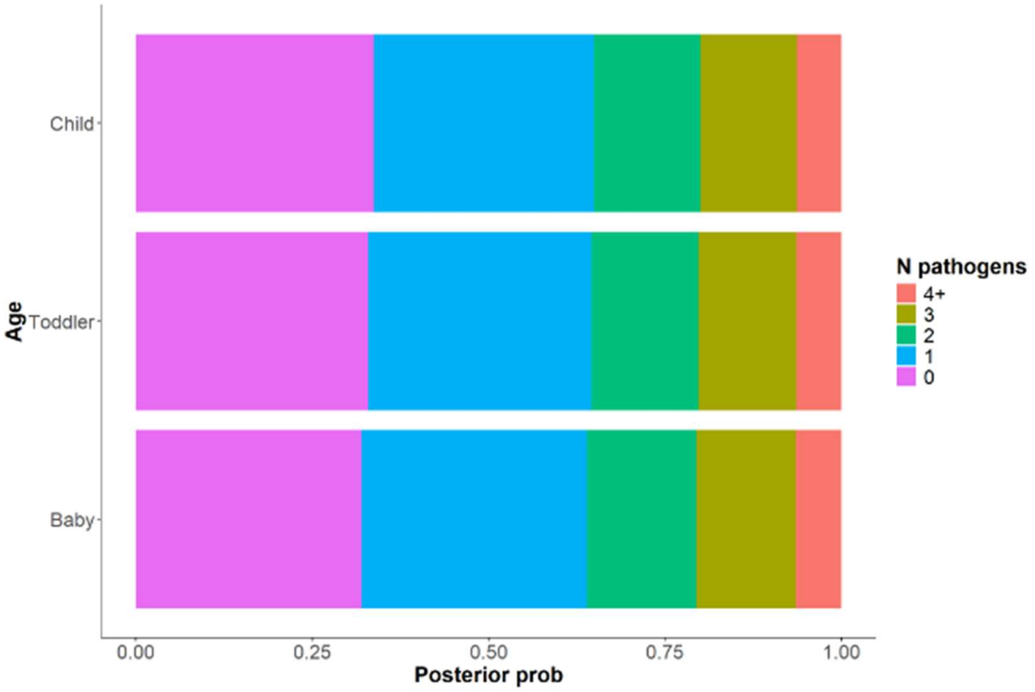

Correlation of Dose

```
exposure_corr =
  exposure_dose_per_hour$B %>%
  as_tibble() %>%
  bind_rows(exposure_dose_per_hour$T %>%
            as_tibble()) %>%
  bind_rows(exposure_dose_per_hour$C %>%
            as_tibble()) %>%
  as.matrix() %>%
  cor(method = "spearman")
rownames(exposure_corr) =
  colnames(exposure_corr) %<>%
  case_match("eaec_aata" ~ "EAEC aata",
             "aeromonas" ~ "Aeromonas",
             "cholera" ~ "Cholera",
             "eaec_aaic" ~ "EAEC aaic",
             "epec_bfpa" ~ "EPEC bfpa",
             "etec_lt" ~ "ETEC LT",
             "epec_eae" ~ "EPEC eae")

corrplot(exposure_corr,
         type = "upper",
         order = "hclust",
         col = viridis_pal()(15),
         method = "ellipse",
         tl.col = "black",
         addCoef.col = 'white')
```

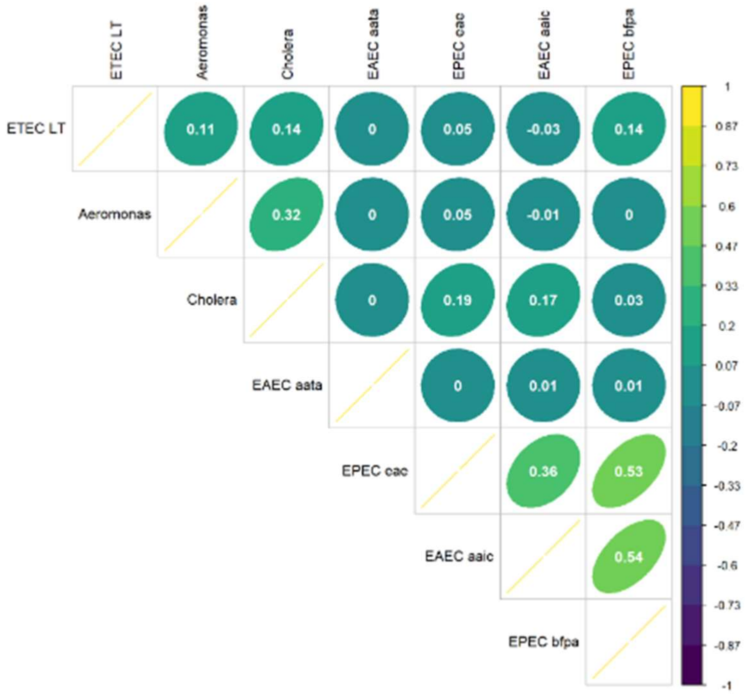
ETEC LT
Aeromonas
Cholera
EAEC aata
EPEC eae
EAEC aaic
EPEC bfpa
0.11
0.14
0
0.05
-0.03
0.14
0.32
0
0.05
-0.01
0
0
0.19
0.17
0.03
0
0.01
0.01
0.36
0.53
0.54
1
0.87
0.73
0.6
0.47
0.33
0.2
0.07
-0.07
-0.2
-0.33
-0.47
-0.6
-0.73
-0.87
-1
