# Supplementary material for: A multi-pathogen behavioral exposure model for young children playing in public spaces in developing communities
Source: PLoS Negl Trop Dis. 2024 Oct 8;18(10):e0012564. doi: 10.1371/journal.pntd.0012564 (PMC11554075; doi:10.1371/journal.pntd.0012564)
Supplement: Attachment — Submitted filename: PNTD-D-24-00258.docx [file pntd.0012564.s004.docx]

**Methods (Limit 20000 Characters)**

-Are the objectives of the study clearly articulated with a clear testable hypothesis stated?

The objectives of the study are clearly articulated, but the hypothesis is not explicitly stated. Though not necessarily a numerical estimate, a written hypothesis regarding the risk of exposure utilizing the presented model may help clarify the purpose and context of the study.

-Is the study design appropriate to address the stated objectives?

The study design is appropriate. This multidisciplinary approach obtains the data necessary to create a model that is a function of environmental availability of pathogens, child behavior, and microbial transfer rates. Though the soil samples were collected during the dry season 7 months prior to the time of child behavior observations during the wet season, this limitation and its potential implications are clearly explored in the Discussion section.

-Is the population clearly described and appropriate for the hypothesis being tested?

The population is described in the Results rather than Methods. Though a prior study is referenced, the authors may consider adding a summary of participant enrollment in the Methods section.

-Is the sample size sufficient to ensure adequate power to address the hypothesis being tested?

No sample size or power calculations are included. The authors may share any pre-analysis power calculations or add post-hoc calculations to the supplemental files. Please note that post-hoc power calculations using observed effect size estimates may be noisy (Gelman 2019, DOI 10.1097/SLA.0000000000003089).

-Were correct statistical analysis used to support conclusions?

A Bayesian multivariate model seemed appropriately employed to support the main conclusions. A Pearson’s rank correlation coefficients were presented to confirm credibility of qPCR results as well. Further detailed review of the manuscript by a biostatistician is needed.

-Are there concerns about ethical or regulatory requirements being met?

There are no significant concerns, but no clear description of the Institutional Review Board approval and consent process are included in this manuscript. These points are outlined in the cited study Medgyesi et al (reference 15), but the authors may consider explicitly summarizing IRB approval and consent in this article as well.

**Results (Limit 20000 Characters)**

-Does the analysis presented match the analysis plan?

The analysis presented matches the analysis plan in the Methods section.

-Are the results clearly and completely presented?

The results are clearly and completely presented through both written descriptions and visualized figures. See minor comment regarding Figure 2 below.

-Are the figures (Tables, Images) of sufficient quality for clarity?

Figures are of sufficient quality for clarity except Figure 2. The caption of Figure 2, as it is currently written, lists three variables (qPCR gene copies/g, *E. coli* cfu/g, and number of pathogen gene targets for a given sample), so it is slightly unclear as to which two variables are represented in the correlation coefficients. It appears that the coefficients in the matrix reflect the correlation between either a gene target from qPCR or *E. coli* cfu/g with other gene target or *E. coli* cfu/g. It may be beneficial to adjust the caption to clarify exactly which two data sets are being correlated.

Conclusions

-Are the conclusions supported by the data presented?

Conclusions are supported by the data presented, though “relatively high likelihood of pathogen exposure for children playing in this peri-urban settlement” (Lines 392-393) would be more informative if put into context. Is it high relative to how much pathogen exposure was expected in this sample or high relative to estimated exposure elsewhere?

-Are the limitations of analysis clearly described?

Limitations are clearly described. Each limitation’s possible impact on the results is explored. The main limitations appear to lead to more conservative estimates of pathogen exposure rather than over-extrapolation of the data.

-Do the authors discuss how these data can be helpful to advance our understanding of the topic under study?

The authors discuss how these data can be helpful to advance the understanding of how children interact with their environment, as well as its implications for pathogen exposure and control in public spaces. The study contributes to the efforts to elucidate the mixed efficacy of household versus public infrastructure interventions. The results also guide future studies toward exploring reasons for the changes in pathogen exposure during development such as behavioral or anatomical changes, implying that WASH interventions may also be customized to the age group of its recipients. The presented model may also be further developed to apply to other types of studies that aim to quantify pathogen exposure as a function of multiple factors.

-Is public health relevance addressed?

Public health relevance is addressed. This study is useful for future studies in modeling pathogen exposure in children playing in public spaces, which can be quantified and adjusted for in future WASH studies.

**Editorial and Data Presentation Modifications? (Limit 20000 Characters)**

Use this section for editorial suggestions as well as relatively minor modifications of existing data that would enhance clarity. If the only modifications needed are minor and/or editorial, you may wish to recommend “Minor Revision” or “Accept”.

I recommend “Minor Revision”. For Figure 2 caption: Suggest clearly listing two variables (qPCR gene copies/g or E. coli cfu/g versus number of pathogen gene targets present for a given sample by qPCR) to clarify which two variables are being assessed for correlations.

**Summary and General Comments (Limit 20000 Characters)**

Use this section to provide overall comments, discuss strengths/weaknesses of the study, novelty, significance, general execution and scholarship. You may also include additional comments for the author, including concerns about dual publication, research ethics, or publication ethics. If requesting major revision, please articulate the new experiments that are needed.

The authors present a model to quantify the probability of enteric pathogen exposure from playing in public spaces in children in a peri-urban settlement in Haiti. This study is significant as pathogen exposure in public is often a variable that is not well-characterized yet is likely impactful during WASH intervention delivery and evaluation. Claims are properly placed in the context of previous literature. The authors outline the limited success of prior WASH trials that focused only on the household, evidence of children’s likely exposure to public spaces, and improvements in health outcomes from trials that delivered interventions for public infrastructure. This study is novel as it quantifies risk of pathogen exposure from public spaces as a function of environmental availability of pathogens, child developmental stage, and microbe transfer rate. The authors present a preliminary model that may be further developed and applied to many other scenarios and data sets. Strengths of the study include: combining microbiological, molecular, and behavioral data to quantify risk of pathogen exposure in a low-resource setting, flexibility of the presented model to apply to different settings and data sets; clear writing; and transparent discussion of limitations. However, its main weakness is the lack of mitigating practices for some limitations. For example, the overestimation of viable pathogenic bacteria (Lines 446-450) may be mitigated by checking for RNA expression of the gene targets in bacterial culture, if possible. If not, the authors may briefly describe how future studies may mitigate such limitations. In Lines 397-399, are there citations from other studies demonstrating the plausibility of the reasons that could explain differences by age? This could strengthen the authors’ points. In lines 476-478, please include citations that would support this statement and expand on this concept further. In endemic areas, in addition to resistance, the immune system may have a more robust immunoprotective or immunoregulatory response versus an immunopathologic response. Or in high contamination environments, chronic infection may blunt the immune response. More nuance is needed here.

**Confidential Comments to Editor (Limit 20000 Characters)**

Comment here if you have any editorial concerns that should be relayed confidentially to the editor and/or journal staff. Comments entered in this field will NOT be communicated to the authors.

None.
